# Halogenated Boroxine K_2_[B_3_O_3_F_4_OH] Modulates Metabolic Phenotype and Autophagy in Human Bladder Carcinoma 5637 Cell Line

**DOI:** 10.3390/molecules29122919

**Published:** 2024-06-19

**Authors:** Nikolina Elez-Burnjaković, Lejla Pojskić, Anja Haverić, Naida Lojo-Kadrić, Maida Hadžić Omanović, Ajla Smajlović, Svetoslav Kalaydjiev, Milka Maksimović, Bojan Joksimović, Sanin Haverić

**Affiliations:** 1 Faculty of Medicine Foča, University of East Sarajevo, Studentska 5, 73 300 Foča, Bosnia and Herzegovina; nikolinaa85@hotmail.com; 2 Institute for Genetic Engineering and Biotechnology, University of Sarajevo, Zmaja od Bosne 8, 71 000 Sarajevo, Bosnia and Herzegovina; lejla.pojskic@ingeb.unsa.ba (L.P.); anja.haveric@ingeb.unsa.ba (A.H.); naida.lojo@ingeb.unsa.ba (N.L.-K.); maida.hadzic@ingeb.unsa.ba (M.H.O.); ajla-rizvanovic@hotmail.com (A.S.); sanin.haveric@ingeb.unsa.ba (S.H.); 3 Agilent Technologies LDA UK Ltd., 5500 Lakeside, Stockport SK8 3GR, UK; svetoslav.kalaydjiev@agilent.com; 4 Faculty of Science, University of Sarajevo, Zmaja od Bosne 33, 71 000 Sarajevo, Bosnia and Herzegovina; mmaksimo@yahoo.co.uk

**Keywords:** cytotoxicity, autophagy, BC cells, gene expression, metabolic phenotype

## Abstract

Halogenated boroxine K_2_[B_3_O_3_F_4_OH] (HB), an inorganic derivative of cyclic anhydride of boronic acid, is patented as a boron-containing compound with potential for the treatment of both benign and malignant skin changes. HB has effectively inhibited the growth of several carcinoma cell lines. Because of the growing interest in autophagy induction as a therapeutic approach in bladder carcinoma (BC), we aimed to assess the effects of HB on metabolic phenotype and autophagy levels in 5637 human bladder carcinoma cells (BC). Cytotoxicity was evaluated using the alamar blue assay, and the degree of autophagy was determined microscopically. Mitochondrial respiration and glycolysis were measured simultaneously. The relative expression of autophagy-related genes BECN1, P62, BCL-2, and DRAM1 was determined by real-time PCR. HB affected cell growth, while starvation significantly increased the level of autophagy in the positive control compared to the basal level of autophagy in the untreated negative control. In HB-treated cultures, the degree of autophagy was higher compared to the basal level, and metabolic phenotypes were altered; both glycolysis and oxidative phosphorylation (OXPHOS) were decreased by HB at 0.2 and 0.4 mg/mL. Gene expression was deregulated towards autophagy induction and expansion. In conclusion, HB disrupted the bioenergetic metabolism and reduced the intracellular survival potential of BC cells. Further molecular studies are needed to confirm these findings and investigate their applicative potential.

## 1. Introduction

Autophagy is a dynamic process, conserved in all eukaryotes. It is responsible for the degradation of cytoplasmic content and the cell’s response to stress, embryonic development, pathogens, and aging. The process is also involved in the pathology of human diseases, such as cancer, diabetes, cardiomyopathy, and neurodegenerative diseases. Among the three types of autophagy, chaperone-mediated autophagy, microautophagy, and macroautophagy, the term autophagy mainly refers to the last one (hereinafter autophagy) [1]. In normal tissues, autophagy is at a low level, and it increases in metabolic stress and in the absence of oxygen and nutrients in tumors. Many cancer types, such as bladder, lung, and colorectal [2,3], as well as melanoma [4], show increased basal autophagy and are associated with aggressiveness and a poor prognosis [5]. Metastatic tumors are highly “dependent” on autophagy, which provides the metabolic energy needed for growth. However, if extensively or inappropriately activated, autophagy can promote apoptosis (type I) or function as an alternative cell death pathway, which is called autophagic cell death (type II). Autophagy can either promote the death of cancer cells or serve as a survival mechanism against apoptotic or necrotic death induced by various anticancer treatments [6]. Given the contradictory role of autophagy during tumor initiation and progression, the therapeutic use of autophagy depends on the context and must be approached individually.

Bladder carcinoma (BC) is one of the most common malignancies, with an increasing incidence worldwide [7]. The majority of new cases are non-muscle-invasive tumors that are commonly treated by radical resection, radiotherapy, and chemotherapy. However, more than half of the superficial bladder cancers will recur, with 10–40% resulting in metastatic outcomes [8].

The non-muscle-invasive bladder carcinoma cell line 5637 has been used as an in vitro model for high-risk superficial bladder tumors with specific molecular characteristics (Bcl-2 and Bcl-X positive expression and *P53* tumor suppressor mutation) [9]. Loss of p53 tumor suppressor function, due to the P53R280T mutation [10,11], promotes the Warburg effect [11]. P53 is known to reduce the glycolysis rate by increasing the activity of fructose-2,6-bisphosphatase. This mechanism is also involved in the regulatory pathways of apoptosis [12]. This mechanism seems to increase oxidative phosphorylation. Earlier studies have confirmed increased glucose and glutamine uptake, along with pyruvate and alanine production, in 5637 cell lines in vitro [13]. Therefore, 5637 cells show inhibited apoptosis, increased cell proliferation, and chemotherapy resistance. Silencing of the *P53* mutant decreases cell viability [14]. High levels of basal autophagy in 5637 cells are confirmed by LC3 immunofluorescence staining in a nutrient-rich environment. The inhibition of basal autophagy results in apoptotic cell loss [3]. The efficacy of chemotherapy is limited because of drug resistance, while autophagy may account for the failure of chemotherapy treatment in BC. Large proportions of patients with advanced BC are resistant to chemotherapy and are characterized by specific molecular signatures [15]. The cooperative inhibition of autophagy is shown to be a vital strategy against cisplatin resistance in human BC cells [15]. To reduce chemoresistance, efforts should focus on several areas. Multi-omics sequencing and analysis can identify reliable biomarkers for predicting chemosensitivity in bladder cancer and might pinpoint the patient groups that are likely to respond to cytotoxic treatments. Targeting dysregulated pathways in chemotherapy-resistant patients could reverse resistance. Additionally, addressing other tumor microenvironment components, such as immune cells and cancer-associated fibroblasts, is crucial. Precision drug delivery using nanotechnology and antibody–drug conjugates shows promise. These strategies aim to improve response rates and increase overall and progression-free survival [16]. Meanwhile, cisplatin-based adjuvant chemotherapy remains a valid option for muscle-invasive bladder cancer [17].

Therapeutic strategies for BC may cause unfavorable side effects and complications. Therefore, autophagy induction is a promising therapeutic approach [15]. The association of the Warburg effect in cancers with tumor-related autophagy may be relevant for the further development of experimental therapeutics and cancer prevention.

In this work, we investigated the potential of dipotassium trioxohydroxytetrafluorotriborate, K_2_[B_3_O_3_F_4_OH], also known as halogenated boroxine (HB), to promote autophagy in BC cell lines. HB is a derivative of the cyclic anhydride of boronic acid [18], containing four fluorine atoms substituted in a six-membered ring. Patented as a prevention and therapeutic agent for various skin changes [19,20], HB has been submitted to numerous in vitro studies [21,22,23,24,25]. It acts as an inhibitor of horseradish peroxidase (HRP) [26], catalase [27], superoxide dismutase [28], and carbonic anhydrases [29]. In the GR-M cell line, HB significantly downregulates the relative expression of *IGF-1*, *hTERT*, and *BCL-2* genes [24,30], suggesting its antitumor activity. At a physiological concentration of Ca^2+^, HB inhibits the growth of tumor cells without having such an effect on normal cells [31]. The anti-tumor activity of HB in vitro has also been proven against mammary carcinoma (4T1), melanoma (B16F10), and squamous cell carcinoma (SCCVII) [32]. HB significantly inhibits tumor growth in vivo, regardless of the means of application [32]. Temporary mild-to-moderate HB dose-dependent lesions were observed in the kidneys and livers of Wistar rats after single intraperitoneal doses of HB at 25, 35, and 45 mg/kg/body weight (bw) in comparison with the control and 10 mg/kg/bw treatments. Adverse effects were not detected in a single-dose administration lower than 35 mg/kg or in repeated, nine-day dosages with 10 mg/kg/bw [33]. The median lethal dose (LD50) of HB in Sprague Dawley and Wistar rats and BALB/c mice ranges from 63 to 75 mg/kg, meaning that HB shows moderate toxicity [34].

According to the previous findings on the toxic effects of HB, we aimed to test if HB may be used as an autophagy inducer in human BC cells and its potential to modify the Warburg effect.

## 2. Results

### 2.1. Cytotoxic Effects of HB on BC Cells

**Figure 1:**
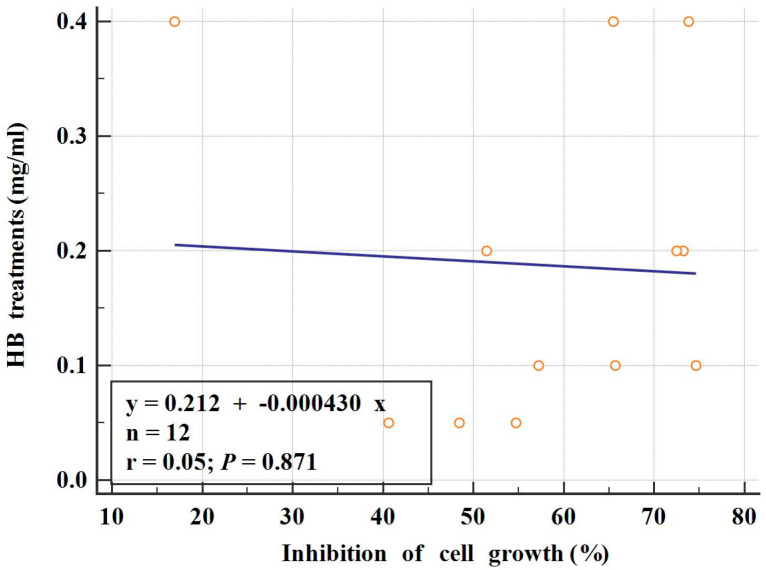
Association between BC cell growth and different HB treatments (simple linear regression; r = 0.05; *p* = 0.871). Cells were treated for 24 h after initial incubation. Cultures without test substances, set as positive control, and negative control cultures, without cells, were used to calculate the percentage of cell growth inhibition (https://tools.thermofisher.com, accessed on 15 August 2023; the alamarBlue^®^ Assay, Thermo Fisher Scientific, Waltham, MA, USA).

The results of the alamar blue assay revealed BC cell growth inhibition of 47.16% at a 0.05 mg/mL HB concentration. HB concentrations of 0.1 and 0.2 mg/mL reduced cell growth to 65.18% and 64.43%, respectively. The inhibition percentage at 0.4 mg/mL was 53.41%. The association between the HB concentration and the percentage of cell growth inhibition was estimated by a simple linear regression calculation (Figure 1).

### 2.2. Evaluation of Autophagy Induction by HB

Autophagosome induction was semi-quantified. Four categories were established according to the estimated number of autophagosome signals per cell. According to the predefined criteria, 200 cells were observed for autophagy markers (signals) for each treatment.

Compared to the negative control, which had a total of 22 cells with autophagy signals, the 0.05 mg/mL HB concentration resulted in 141 cells with autophagy signals, with most cells having 5–10 detected autophagosomes. At 0.1 and 0.2 mg/mL HB treatments, autophagy markers were detected in 166 and 148 cells, respectively. At a 0.1 mg/mL HB concentration, a higher frequency of cells with 5–10 signals was found compared to the 0.2 mg/mL HB treatment, where more cells with coupled signals were detected. At a 0.4 mg/mL HB concentration, 141 cells were positive for autophagy, with the highest frequency of cells with the coupled signals. Autophagy was successfully induced in the positive control, where 191 cells had different types of autophagy signals.

**Figure 2:**
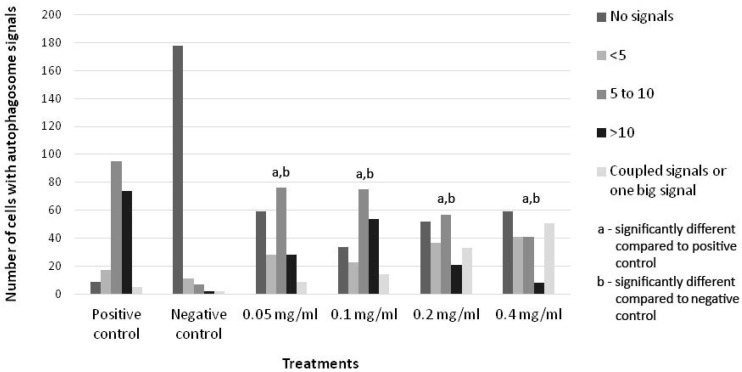
Distribution of frequency of detected autophagosome signals; positive (+) control—starvation-induced autophagy cells; negative (−) control—untreated cells. Four categories were established according to the estimated number of autophagosome signals per cells. According to predefined criteria, 200 cells were observed for autophagy markers (signals) for each treatment. Differences for all treatments and quantification of autophagosome signals were significant (*p* < 0.05).

**Figure 3:**
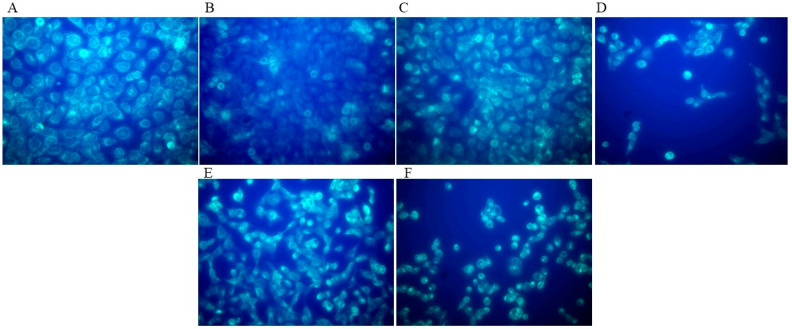
Autophagy detection using Autophagy Assay kit (Sigma-Aldrich, St. Louis, MO, USA). Epifluorescence detection was performed using a BX51 microscope with 400× magnification (Olympus, Tokyo, Japan); the degree of autophagy was photo-documented with a DP50 camera analyzed using View Finder Lite software version 1.0.143c (Olympus, Japan). (**A**)—positive control; (**B**)—negative control; (**C**)—0.05 mg/mL treatment; (**D**)—0.1 mg/mL treatment; (**E**)—0.2 mg/mL treatment; and (**F**)—0.4 mg/mL treatment.

Different categories of autophagosome induction were observed in all cultures treated with HB in concentrations ranging from 0.05 to 0.4 mg/mL. Autophagy was successfully induced in the positive control, while the negative control showed low levels of basal autophagy according to the detection of autophagosome signals (Figure 2 and Figure 3, Supplementary Materials).

Comparisons of detected autophagosome signals compared to the negative control were statistically significant in 0.05, 0.1, 0.2, and 0.4 mg/mL HB treatments (*p* < 0.05). Comparisons of autophagosome signal ratios, compared to the positive control, were statistically significant at all tested HB concentrations (*p* < 0.05).

### 2.3. Cellular Energy Phenotype and the Investigation of Metabolic Switching: OCR and ECAR Rates in Response to HB Treatment—Nts

The XF Cell Energy Phenotype test was performed for glycolysis and mitochondrial respiration determination. The oxygen consumption rate (OCR), as a measure of mitochondrial respiration, and the extracellular acidification rate (ECAR), as a measure of glycolysis, were determined in baseline and stressed phenotypes at 0.05, 0.1, 0.2, and 0.4 mg/mL HB concentrations in both positive and negative controls.

**Figure 4:**
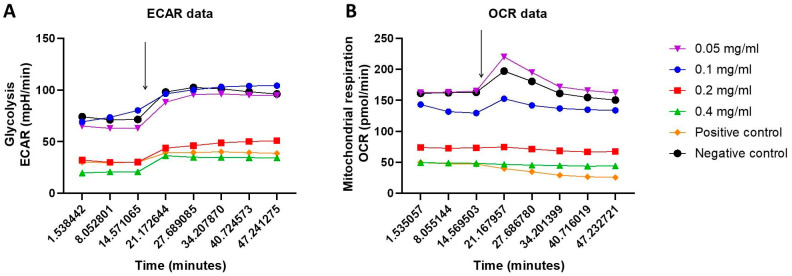
Phenotype Stress Test assessment of basal (three initial measurements) and stressed (five subsequent measurements) metabolic rates in BC cells seeded at 4 × 10^4^/well: (**A**) glycolysis (ECAR) and (**B**) mitochondrial respiration (OCR) were measured on a Seahorse XF HS Mini Analyzer. ECAR—extracellular acidification rate; OCR—oxygen consumption rate. Results are presented as mean values of duplicate measurements.

Significant differences between basal and stressed OCR values, tested by the Mann–Whitney test, were registered only for the positive control (*p* = 0.0002), with a decrease in the OCR values in a stressed state. ECAR values increased in a stressed state for all treatments. The differences were significant for all HB treatments except for 0.4 mg/mL (*p* = 0.12). There is a clear differentiation in OCR and ECAR values between the negative control, 0.5 and 0.1 mg/mL HB treatments on one side, the highest tested HB concentrations (0.2 and 0.4 mg/mL), and the positive control on the other side (Figure 4).

**Figure 5:**
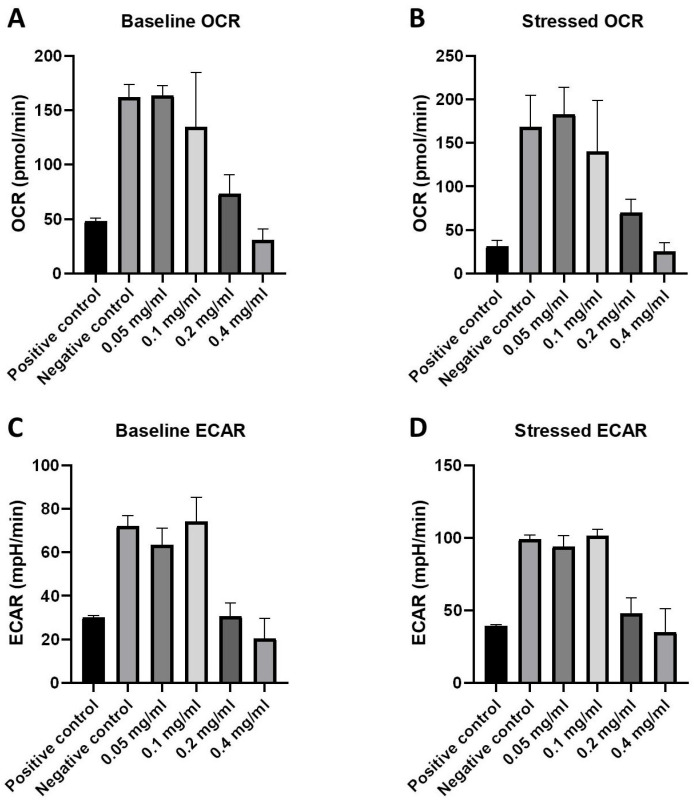
Phenotype Stress Test showing basal and stressed mitochondrial (**A**,**B**) and glycolytic activity (**C**,**D**) of the BC cells. Data are presented as means with SD analyzed by Kruskal–Wallis test, *p* < 0.001. ECAR—extracellular acidification rate; OCR—oxygen consumption rate.

When baseline and stressed OCR and ECAR values were independently compared, significantly higher values (*p* < 0.05; Kruskal–Wallis test) were found for the negative control, and lower HB concentrations (0.05 and 0.1 mg/mL) were found compared to the positive control and higher HB concentrations (0.2 and 0.4 mg/mL) (Figure 5).

### 2.4. Deregulation of Autophagy-Associate Genes by HB

**Figure 6:**
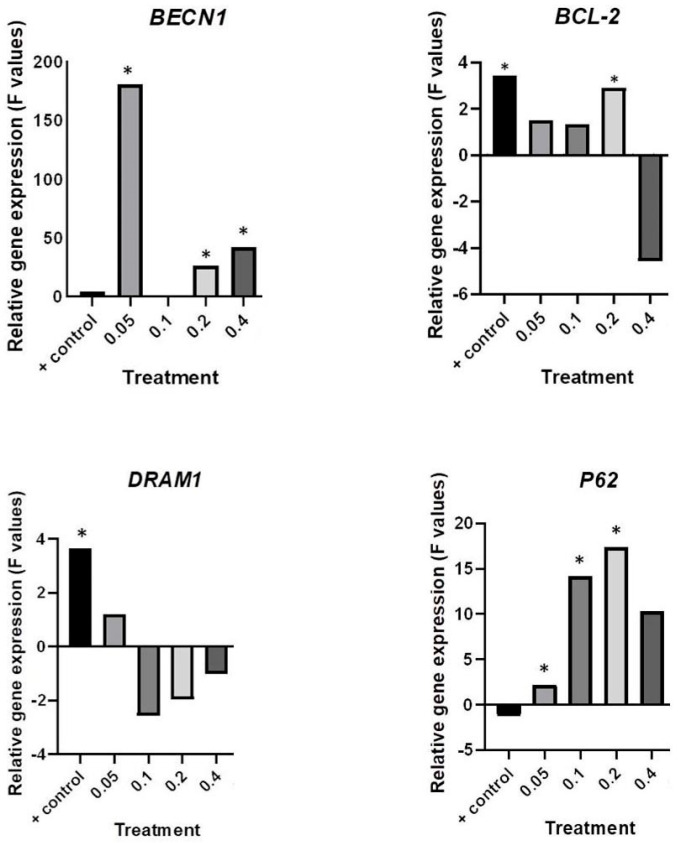
Results of *BECN1*, *BCL-2*, *P62*, and *DRAM1* relative gene expression analysis normalized with the *GAPDH* gene compared with HB-untreated cells (negative control). The F values are shown; * *p* < 0.05.

**Figure 7:**
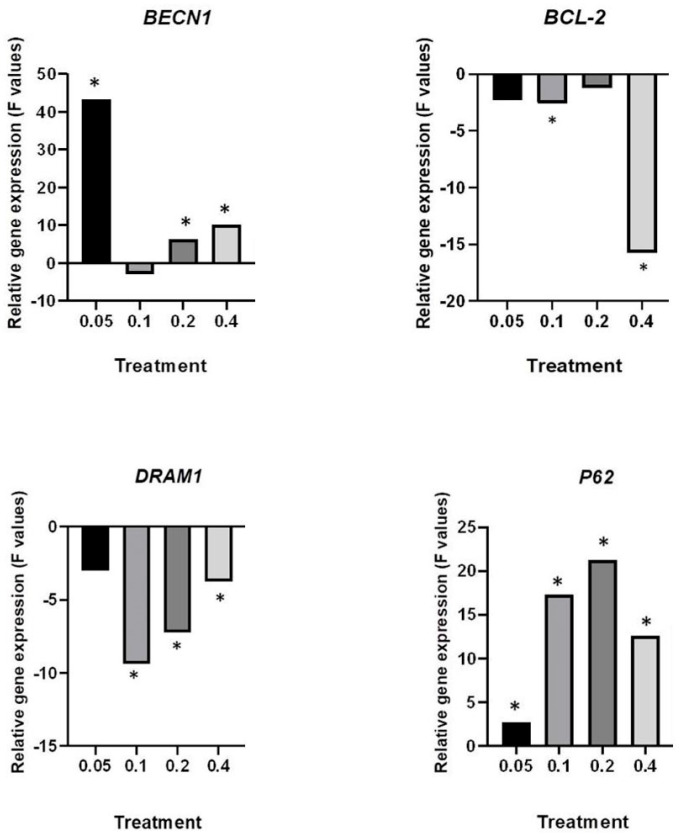
Results of *BECN1*, *BCL-2*, *P62*, and *DRAM1* gene expression analyses normalized with the GAPDH gene were compared with the positive control (starvation-induced autophagy cells). The F values are shown; * *p* < 0.05.

The effects of different HB concentrations on cell cycle deregulation were evaluated using a relative gene expression assay for autophagy markers: *BECN-1*, *BCL-2*, *P62*, and *DRAM1*. The normalized gene expression ratios for all evaluated treatments were compared against corresponding values for negative (untreated cells) and positive controls (Figure 6 and Figure 7).

When compared with the negative control, significant upregulation of *P62* was detected for all except for the 0.4 mg/mL HB concentration. *BECN1* was significantly upregulated in all HB treatments, with unsignificant changes at a 0.1 mg/mL HB concentration. Significant downregulation of *DRAM1* was observed in 0.1–0.4 mg/mL HB treatments compared to the positive control, while *BCL-2* was significantly downregulated at 0.1 and 0.4 mg/mL HB treatments compared to the positive control.

## 3. Discussion

Previous in vitro studies of the anti-proliferative effects of HB have revealed a selective cytotoxic effect on tumor cells and a weak cytotoxic effect on non-tumor cells [21,24,31,32,35]. In this study, we found dose-dependent HB inhibition of cell proliferation. The lowest tested HB concentration (0.05 mg/mL) slightly inhibited BC cell growth, while concentrations of 0.1, 0.2, and 0.4 mg/mL were highly cytotoxic.

Autophagy is a dynamic and complex process that is regulated by multiple molecules and has a contradictory role in tumor metabolism. In BC cells, high basal and protective autophagy against anticancer treatments [15] facilitates the avoidance of apoptotic cell death. Detected autophagosomes in cells treated with different HB concentrations (0.05–0.4 mg/mL) confirm HB-induced autophagy. The level of HB-induced autophagy was lower than in the positive control, where autophagy was successfully induced by starvation. In the negative control, a lower level of basal autophagy was registered. Therefore, HB effects on autophagy induction in BC cells, together with the inhibition of cell proliferation, indicate its high antitumor potential.

Cells possess two energy-producing pathways: mitochondrial respiration and glycolysis. Using the Seahorse XF HS Mini analyzer (Agilent, Santa Clara, CA, USA), we simultaneously measured both of these pathways in live cells, interrogating key cellular functions. Our data showed that starving BC cells (positive control) and 0.2 and 0.4 mg/mL HB treatments exhibit reduced mitochondrial respiration, indicating compromised or dysfunctional mitochondria, as compared to untreated cells and HB treatments at lower concentrations. Untreated BC cells and those treated with HB at lower concentrations showed high basal and stress glycolysis, indicating the ability to use that metabolic pathway as an energy source. These results suggest that HB disrupts glycolysis and reduces the intracellular survival potential of BC cells. Cancer cell metabolism, characterized by the Warburg effect, results in lactate production by aerobic glycolysis. Lactate production generates an acidic and hypoxic microenvironment, which promotes inflammation, tumorigenesis, invasion, and metastasis. Increased OXPHOS increases the production of reactive oxygen species (ROS) and the activation of c-JUN N-terminal kinases (JNK), resulting in autophagy induction. Hypoxia also induces the expression of a hypoxia-inducing factor (HIF-1α), which leads to the inhibition of the mammalian target of rapamycin (mTOR) and autophagy induction [36,37]. The hybrid glycolysis/OXPHOS metabolic state is suitable for the survival of invasive, aggressive tumors. It offers increased metabolic flexibility in cancer cells, which are able to utilize different nutrients in response to increased energy demands due to high proliferation and maintenance of ROS at a moderate level. It promotes genomic instability, which stimulates tumorigenesis and metastasis, and decreases susceptibility to anticancer therapy [38]. Our data strongly suggest that BC cells have a strong hybrid glycolysis/OXPHOS phenotype, which can be interrupted by HB in specific concentrations.

The HB effects on autophagy induction were considered at the gene transcription level as well. HB decreased *DRAM1* gene expression in comparison with the positive control, and it significantly increased the relative gene expression of *P62* in all applied concentrations. DRAM1 stimulates autophagosome elimination [39]. Knock-down of *DRAM1* inhibits P62 binding to the autophagosome, resulting in a decrease in autophagosome degradation. However, the same authors did not report DRAM1’s role in starvation and mTOR-mediated autophagy [40]. Epi-fluorescent analyses of autophagosomes at given concentrations showed the presence of active autophagy, which is supported by increased expression of *BECN1*. Beclin 1 is responsible for the nucleation and maturation of the autophagosomes. The most significant upregulation of *BECN1* was found at 0.05 mg/mL of HB, along with a decreased number of viable cells. An increased frequency of autophagosomes and cells’ degradation may also indicate a blocked autophagy flux [41]. This is supported by increased *P62* expression, which indicates higher requirements for the selective labeling of degradation contents.

One of the main mechanisms of autophagy control is the interaction between the autophagy protein beclin 1 and anti-apoptotic members of the Bcl-2 family (BCL-2, BCL-XL, and MCL-1). Under normal conditions, BCL-2 inhibits beclin 1, and during stress, they separate and further stimulate autophagy. BCL-2 is an anti-apoptotic and anti-autophagy protein [42]. Increased expression of *BCL-2* is present in various types of tumors, including bladder cancer, supporting its tumorigenesis. It is associated with a poor prognosis. In bladder cancer, inhibiting *BCL-2* expression reduces cell proliferation and increases the sensitivity of cells to chemotherapy [43]. Therefore, the regulation of *BCL-2* gene expression is the focus of anti-tumor research. Our results showed a downregulation of *BCL-2* in cells treated with HB at all tested concentrations compared to cells with starvation-induced autophagy. Because a decrease in *BCL-2* expression results in the reduced proliferation of tumor cells [43], HB should be considered a suitable agent for reducing the expression of BCL-2 in bladder tumors. Patterns of relative gene expressions, found in our study, support the hypothesis that the efficiency of HB was associated with the expression of autophagy-specific genes. It has been proven that HB at the same tested concentrations (0.2 and 0.4 mg/mL) reduces BCL-2 expression in UT-7 human leukemia cells [44]. Previously, HB has been shown to reduce *IGF-1* and *hTERT* gene expression [24]. Increased expression of both genes has been related with the poor prognosis in patients with cancer disease [45,46]. The inhibition of telomerase activity and IGF signaling, together with decreased *BCL-2* expression, are possible mechanisms explaining the reduced resistance to cell death. In our study, the results of gene expression and the detection of autophagy showed an increased presence of autophagy. It was previously explained that excessive autophagy can serve as a mechanism of cell death by promoting apoptosis or autophagy cell death due to excessive cell degradation [47]. Although autophagy allows the cell to overcome stress and maintain homeostasis [48], the constant activation of autophagy with a decrease in *BCL-2* expression, observed in BC cells treated with HB, reduced the cell’s ability to survive by causing cell death.

This study has revealed HB inhibition of cell growth. A decreased percentage of BC cell growth at higher HB concentrations can be explained by the intricate interplay between extracellular nucleotides affecting the cell cycle and autophagy modulation in response to cell density, ultimately influencing cell proliferation rates [49,50], as the results of this work have suggested that HB induces autophagy in bladder carcinoma (BC) cells. Additionally, HB targeted both aerobic glycolysis and mitochondrial respiration in BC cells in vitro. By causing significant degradation, metabolic disruption, and downregulation of *BCL-2*, HB increases the cells’ susceptibility to autophagy.

## 4. Materials and Methods

### 4.1. Dipotassium Trioxohydroxytetrafluorotriborate, K_2_[B_3_O_3_F_4_OH], Halogenated Boroxine (HB)—Synthesis and Solution Preparation

HB is a water-soluble white powder. It was synthesized in the Laboratory for Physical Chemistry, Department of Chemistry, Faculty of Science, University of Sarajevo, as previously described [21], with 99.99% purity. The K_2_(B_3_O_3_F_4_OH) stock solution was prepared by dissolving 20 mg of K_2_(B_3_O_3_F_4_OH) in 1 mL of RPMI-1640 culture media.

### 4.2. Cell Culture Treatment

The human urinary bladder carcinoma (BC) 5637 (ATCC, Manassas, VA, USA; Cat. No. ATCC HTB-9) was obtained through collaboration with ICGEB Trieste (Trieste, Italy). Cells were grown in RPMI-1640 media (Sigma-Aldrich, St. Louis, MO, USA) with 10% fetal bovine serum (FBS) supplemented with L-glutamine and antibiotics in a humidified atmosphere of 5% CO_2_ at 37 °C. After the initial 24 h, cell cultures were treated with HB at final concentrations of 0.05, 0.1, 0.2, and 0.4 mg/mL and incubated for an additional 24 h. Untreated tumor cells were used as autophagy negative controls, while for the autophagy positive controls, starvation-induced (serum-free RPMI-1640; Sigma-Aldrich, St. Louis, MO, USA) autophagy cells were used.

### 4.3. Alamar Blue Assay

Cell proliferation was assessed by an alamar blue assay (Invitrogen, Waltham, MA, USA). Each treatment was carried out in triplicate in the 96-well plate, with 10,000 cells per well seeded. Cells were treated with HB for 24 h after the initial incubation. Alamar blue dye was added in an amount equal to 10% of the culture volume 2 h prior to absorbance measuring. Cultures without test substances were set as positive assay controls, while negative assay controls did not contain cells. Medium was used as a blank. Absorbance was measured at 570 and 620 nm using a Multiscan FC plate reader (Thermo Ficher Scientific, Vantaa, Finland). The percentage of cell growth inhibition was calculated according to manufacturer instructions (https://tools.thermofisher.com, accessed on 15 August 2023; The alamarBlue^®^ Assay).

### 4.4. Autophagy Detection

**Figure 8:**
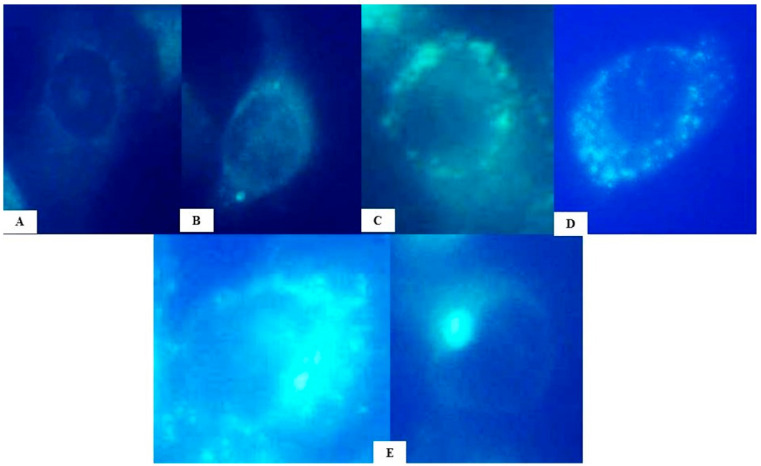
Evaluation of autophagy levels due to the presence and number of signals: no signal (**A**); 0–5 signals (**B**); 5–10 signals (**C**); >10 signals (**D**); and coupled signals or one big signal (**E**). Epifluorescence detection was performed using a BX51 microscope with 1000× magnification (Olympus, Tokyo, Japan).

The Autophagy Assay kit (Sigma-Aldrich, St. Louis, MO, USA) was used to monitor the degree of autophagy using a fluorescent autophagosome marker that specifically binds to autophagosomes. According to the manufacturer’s recommendations, 24 h after incubation of BC cells with HB, epifluorescence detection was performed using a BX51 microscope (Olympus, Japan). The degree of autophagy was photo-documented with a DP50 camera, analyzed using View Finder Lite software (Olympus, Japan), and categorized according to the number of detected signals: no signal; 0–5 signals; 5–10 signals; >10 signals; and coupled signals or one big signal. A total of 200 cells were analyzed per HB treatment or control (Figure 8).

### 4.5. Oxygen Consumption Rate and Extracellular Acidification Rate

BC cells were seeded into Seahorse XF cell culture microplates (4 × 10^4^ cells/well) and incubated overnight. After washing the cells with Agilent Seahorse XF Base Medium, the oxygen consumption rate (OCR) and extracellular acidification rate (ECAR) were measured on a Seahorse XF HS Mini Analyzer (Agilent Technologies, Santa Clara, CA, USA). OCR in pmol/min reveals the rate of decrease in oxygen in the assay medium and, as such, is a measure of mitochondrial respiration in the cells. ECAR in mpH/minute illustrates the rate of increase in proton concentration (or decrease in pH) in the assay medium and reflets changes due to glycolysis. Alterations in metabolic function under basal and “stress” conditions were evaluated using the XFp Cell Energy Phenotype Test Kit (Cat. No. 103275-100; Agilent Technologies), according to the manufacturers’ instructions. Briefly, for the measurement of basal metabolic rates, measurements of OCR and ECAR were taken at the starting assay conditions. To evaluate metabolic rates during increased energy demand, “stress” conditions were induced by the addition of a mix of oligomycin (an ATP synthase inhibitor) and the mitochondrial uncoupler carbonyl cyanide 4-[trifluoromethoxy] phenylhydrazone (FCCP). The data were analyzed using Seahorse Analytics software version 3.0.0.41 (Agilent Technologies, Santa Clara, CA, USA).

### 4.6. q-PCR and Gene Expression Analysis

Five cell populations used in the experiment (untreated BC cells as a negative control and four HB treatments at concentrations of 0.05, 0.1, 0.2, and 0.4 mg/mL) were subsequently analyzed. The total RNA was extracted from harvested cells using an RNA isolation kit (Nucleo Spin RNA, Macherey-Nagel, Düren, Germany). The quantity of isolated RNA was estimated by an RNA fluorometric assay using Qubit 2.0 (Life Technologies, London, UK). For cDNA synthesis, total RNA in a 40 ng/µL concentration was used. High-capacity cDNA Reverse Transcription Kits (Applied Biosystems, Foster City, CA, USA) were used for the generation of cDNA, according to the manufacturer’s instructions.

Amplifications of target genes *BECN1* (beclin 1), *P62/SQSTM* (sequestosome 1), *BCL-2* (B-cell lymphoma 2), and *DRAM1* (Damage-Regulated Autophagy Modulator 1) in replicates were performed using ABI 7300 real-time PCR (Applied Biosystems, USA). The primer sequences are given in Table 1.

The expression of target genes was monitored and normalized with the housekeeping *GAPDH* gene in REST software, version 2 [51], which was also used to compare gene expression at the transcription level in HB treatments and the control. Subsequently, the expression ratio results of the four investigated transcripts were tested for significance (*p* < 0.05) by a randomization test.

### 4.7. Statistical Analysis

To determine if HB-induced autophagy is statistically significant compared to untreated samples and starvation-induced autophagy, a Chi square test was applied (PAST 3.15).

Relative gene expression analysis was evaluated using REST-384, version 2, based on a pairwise fixed reallocation randomization test [51]. The test compares the Ct values of the control and the sample (target gene).

To determine OCR and ECAR significance between baseline and stressed conditions for every treatment, a Mann–Whitney test for independent samples was applied. The Kruskal–Wallis test was applied to determine significance between baselines and stressed conditions, followed by a Conover post hoc analysis.

The Pearson correlation coefficient was calculated to correlate ECAR and OCR baselines with the stressed condition. MedCalc 18.9. software was used, and the significance level was set at 0.05.

## 5. Conclusions

The results of the study suggest autophagy as a cellular and molecular pathway of HB activity in BC cells. This work confirms that HB targets aerobic glycolysis and mitochondrial respiration in BC cells in vitro. Inducing massive degradation, metabolic interruption, and downregulation of *BCL-2* in BC cells HB increases their susceptibility to autophagy. Further studies are needed to elucidate signaling molecules and cellular pathways of HB in autophagy induction in different tumors. The potential of HB based anti-tumor drugs should be additionally investigated.

## Figures and Tables

**Table 1:** Primer sequences for the target genes.

Gene	Reference Sequence (Fasta Acc Number)	Primers
*BECN1*	NM_003766.5	Forward: CTCCCGAGGTGAAGAGCATC
		Reverse: GGGGGATGAATCTGCGAGAG
*P62/SQSTM*	NM_003900.5	Forward: CCGTGAAGGCCTACCTTCTG
		Reverse: TCCTCGTCACTGGAAAAGGC
*BCL-2*	NM_000633.3	Forward: GGGGTCATGTGTGTGGAGAG
		Reverse: GAAATCAAACAGAGGCCGCA
*DRAM1*	NM_018370.3	Forward: TTGGTGCAGCCACGATGTAT
		Reverse: ACACCACAGACAAAGGCCAA

## Data Availability

The data presented in this study are available in article.

## Supplementary Materials

The following supporting information can be downloaded at: https://www.mdpi.com/article/10.3390/molecules29122919/s1.
