# Supplementary figures and images for: Halogenated Boroxine K2[B3O3F4OH] Modulates Metabolic Phenotype and Autophagy in Human Bladder Carcinoma 5637 Cell Line
Source: Molecules. 2024 Jun 19;29(12):2919. doi: 10.3390/molecules29122919 (PMC11206502; doi:10.3390/molecules29122919)

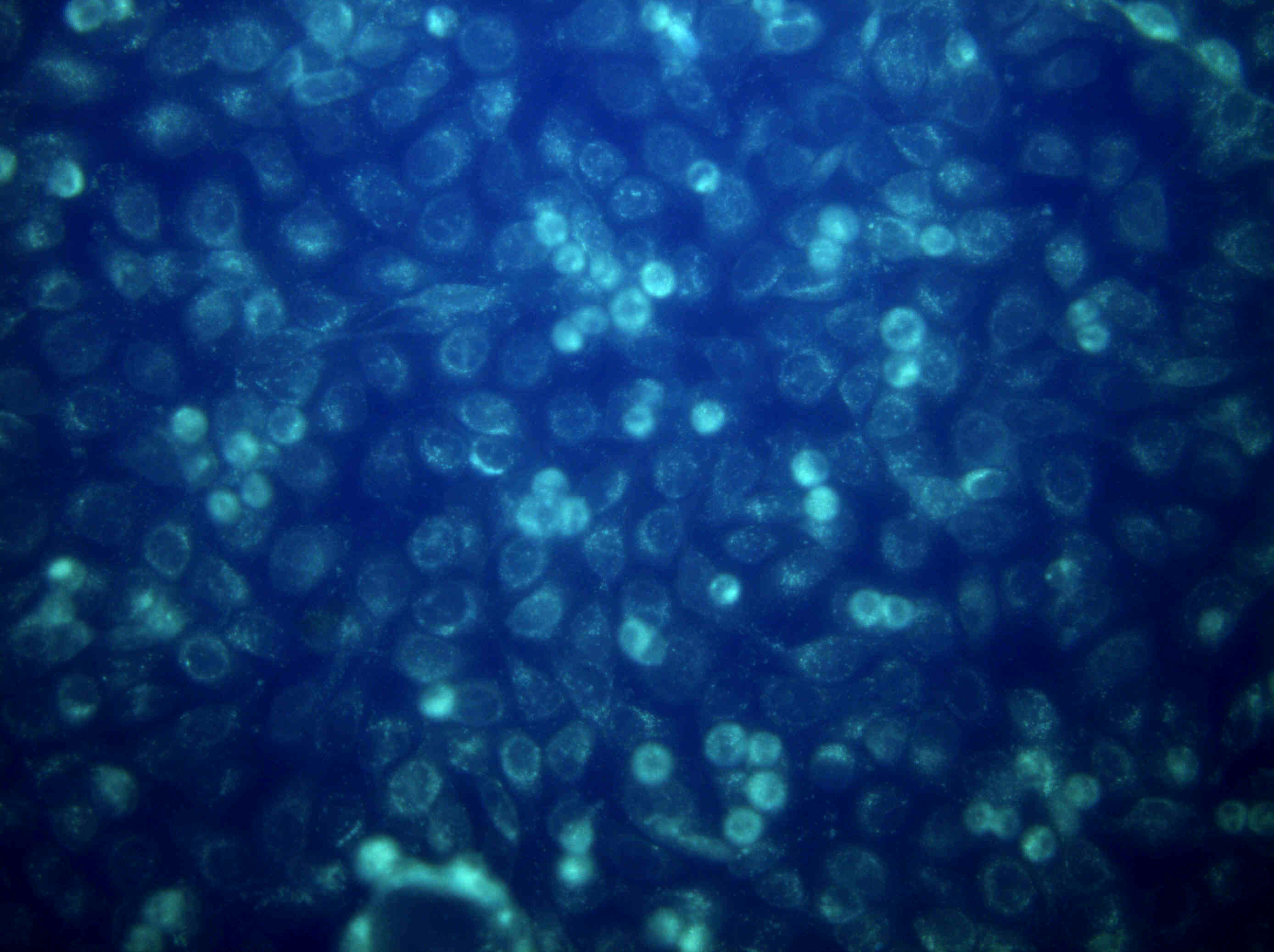

Supplement: Supplementary file 1 [file molecules-29-02919-s001.zip › Supplementary folder 1/0,05 treatment/Figure S1D.JPG]

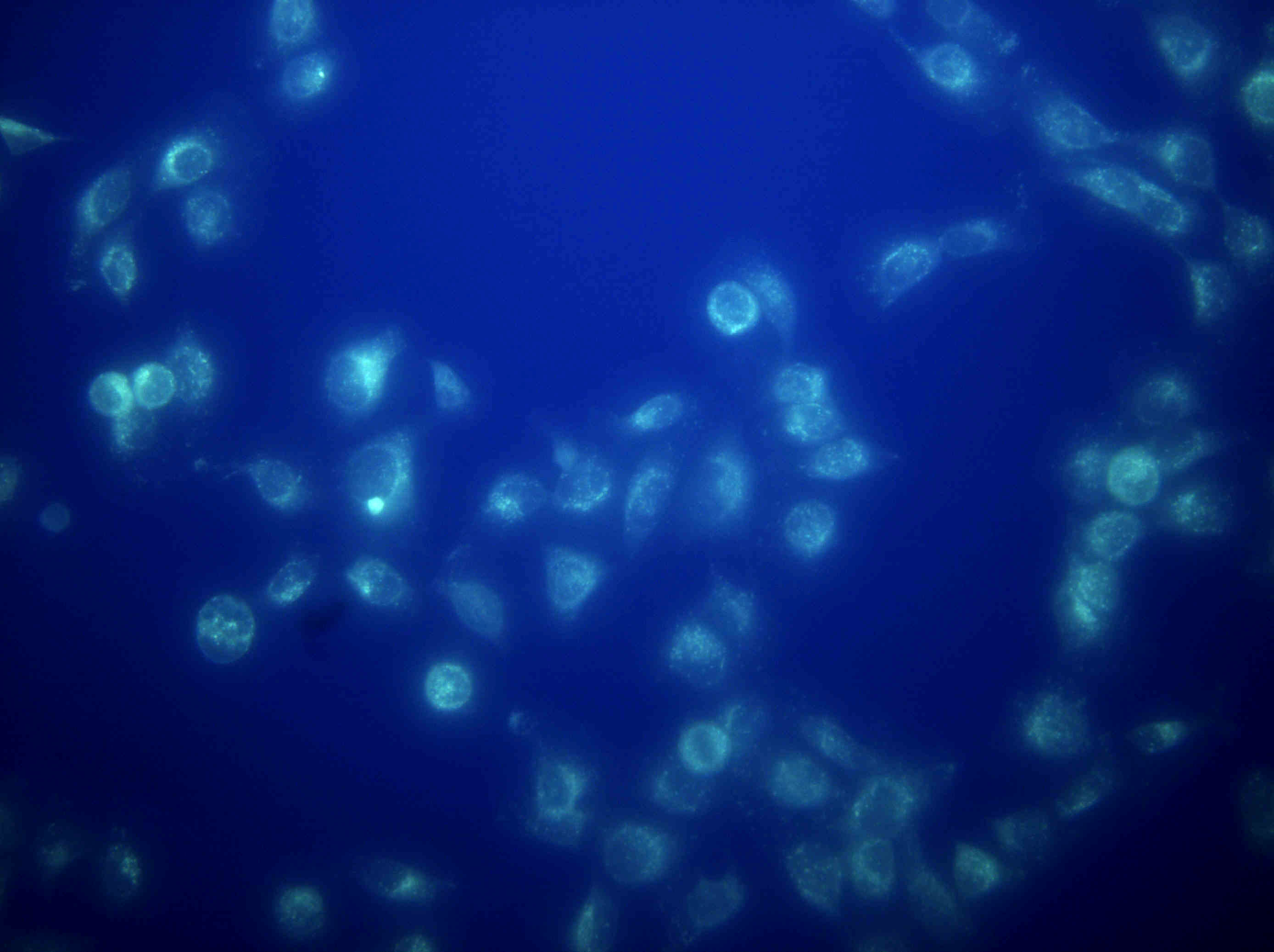

Supplement: Supplementary file 1 [file molecules-29-02919-s001.zip › Supplementary folder 1/0,05 treatment/Figure S2D.JPG]

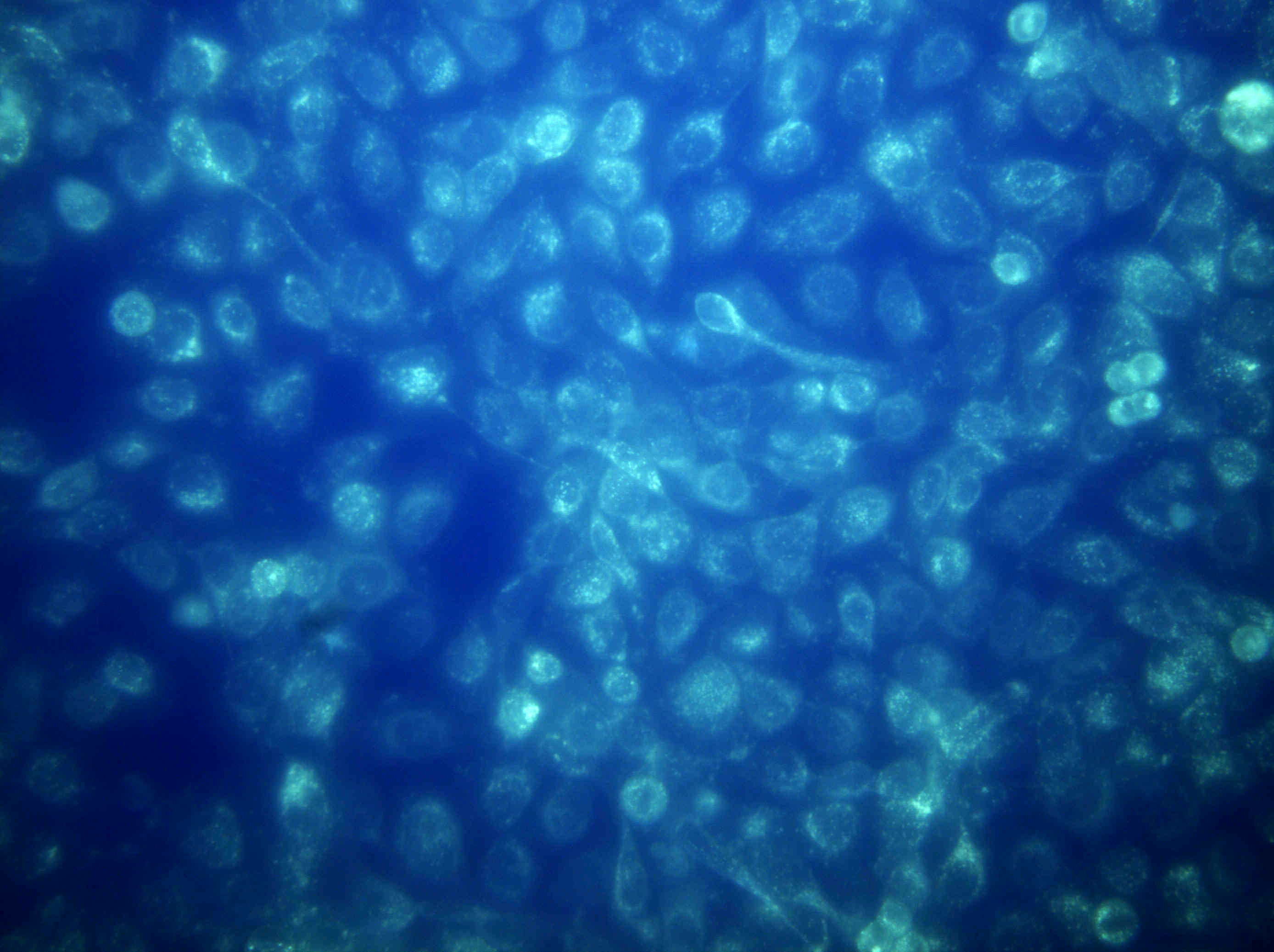

Supplement: Supplementary file 1 [file molecules-29-02919-s001.zip › Supplementary folder 1/0,05 treatment/Figure S3D.JPG]

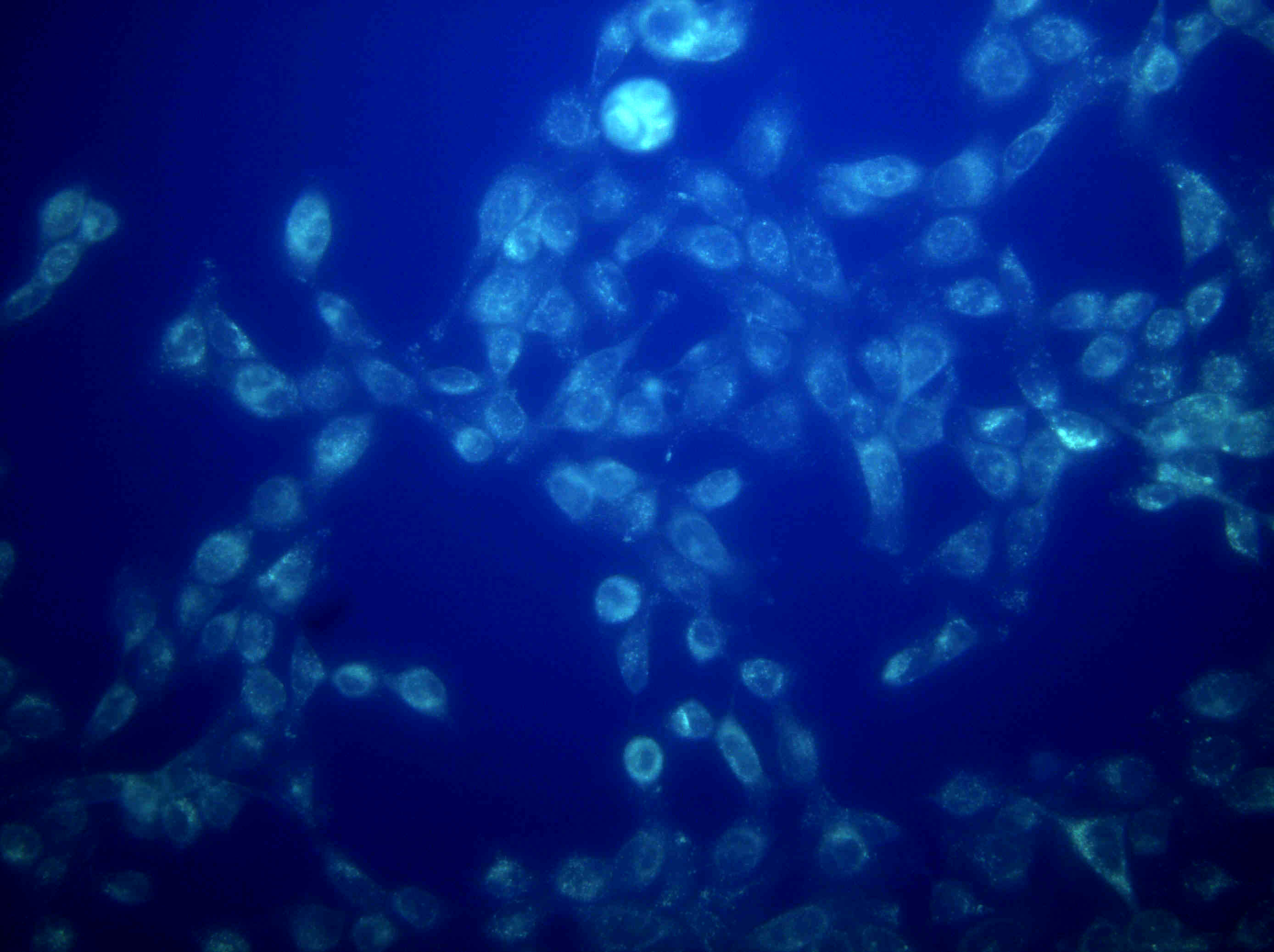

Supplement: Supplementary file 1 [file molecules-29-02919-s001.zip › Supplementary folder 1/0,1 treatment/Figure S1A.JPG]

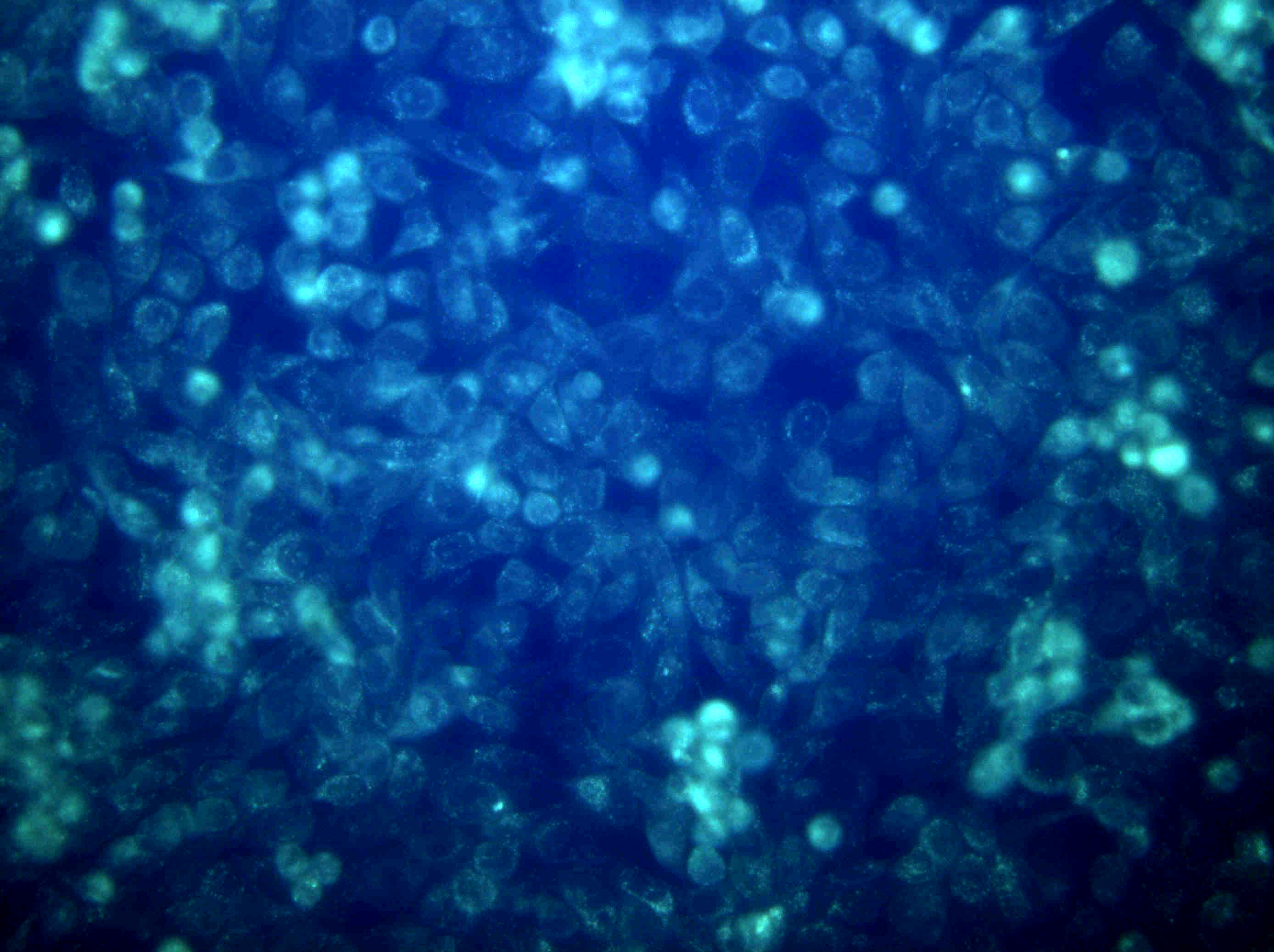

Supplement: Supplementary file 1 [file molecules-29-02919-s001.zip › Supplementary folder 1/0,1 treatment/Figure S2A.JPG]

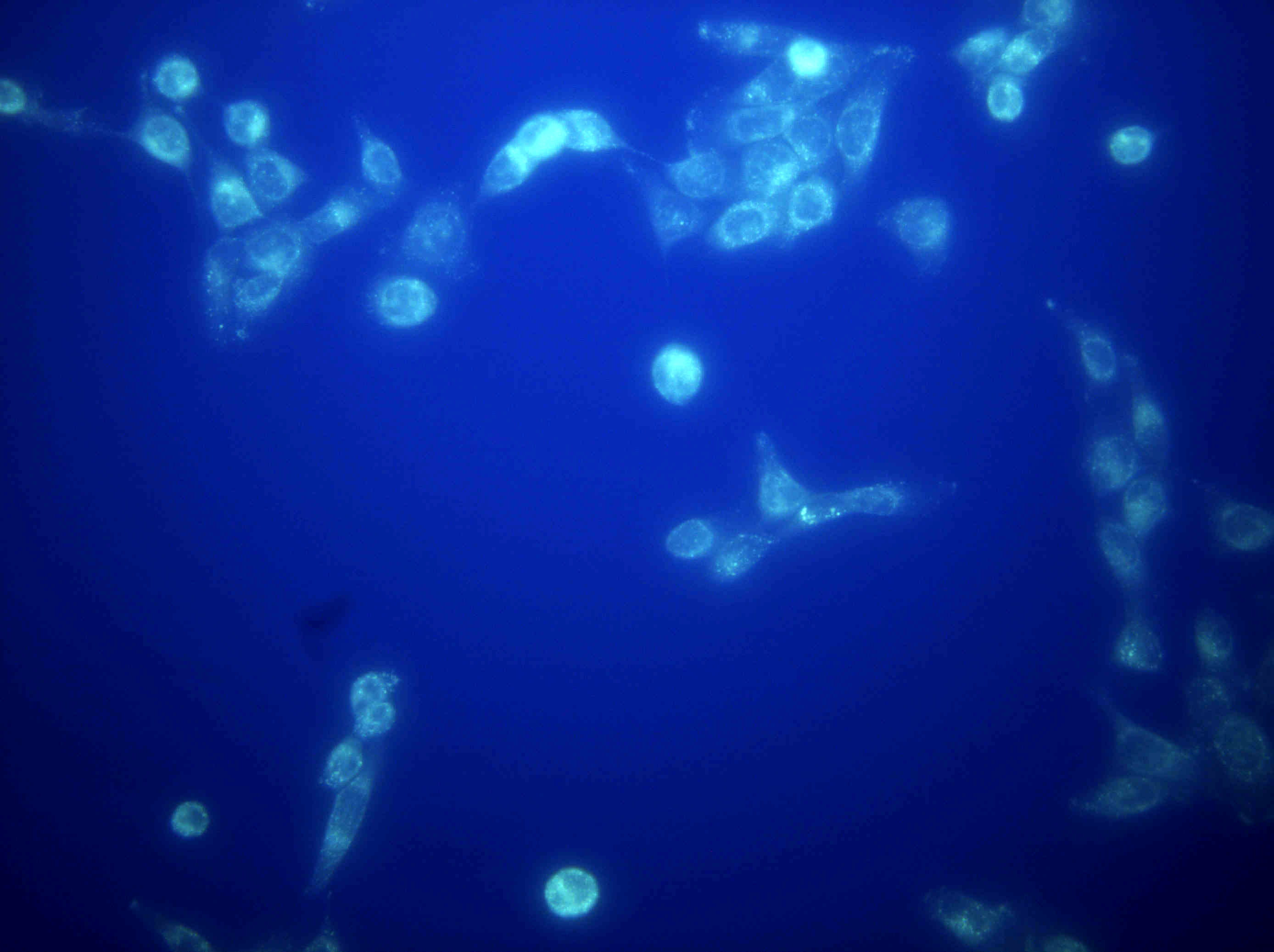

Supplement: Supplementary file 1 [file molecules-29-02919-s001.zip › Supplementary folder 1/0,1 treatment/Figure S3A.JPG]

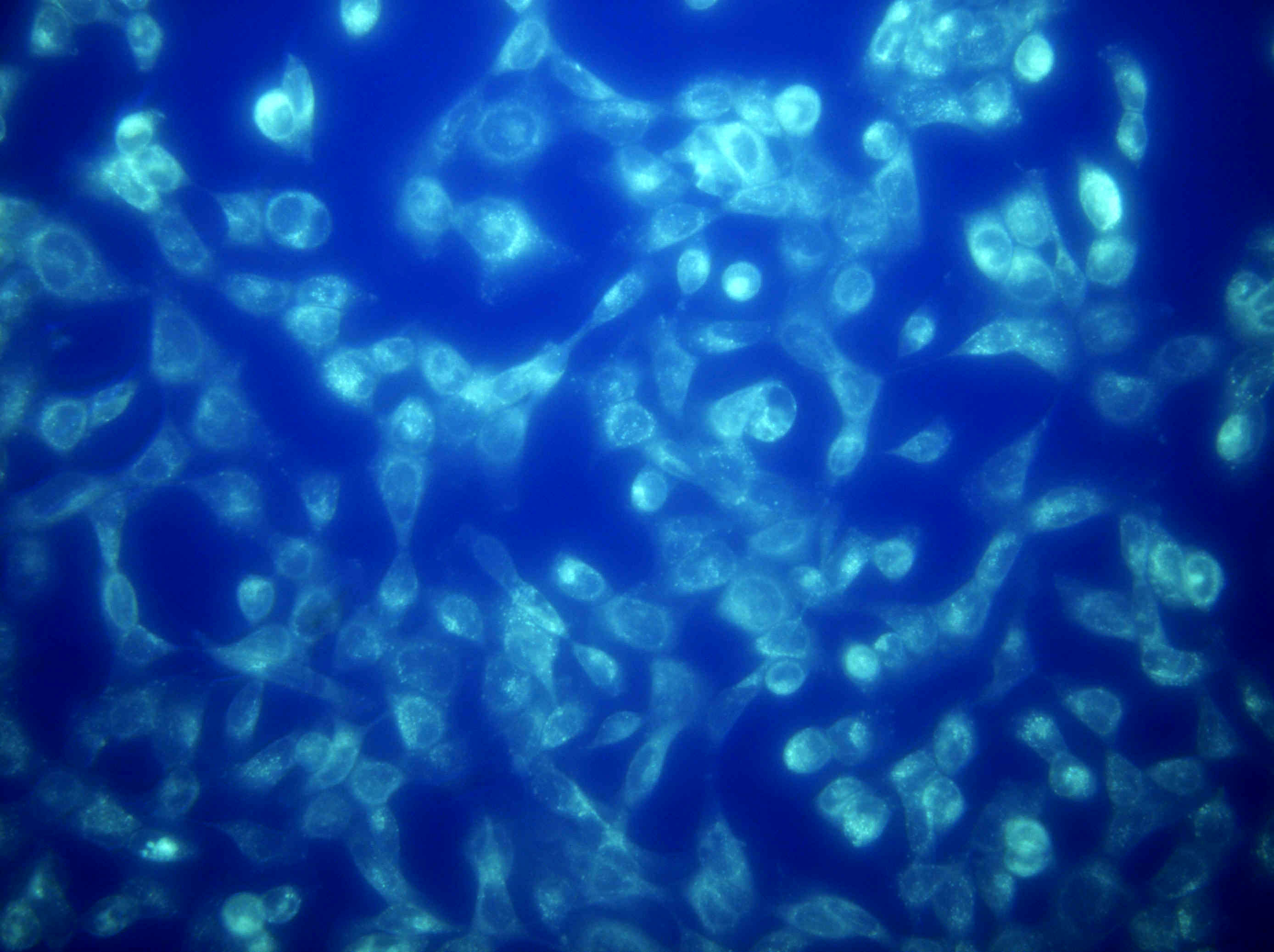

Supplement: Supplementary file 1 [file molecules-29-02919-s001.zip › Supplementary folder 1/0,2 treatment/Figure S1B.JPG]

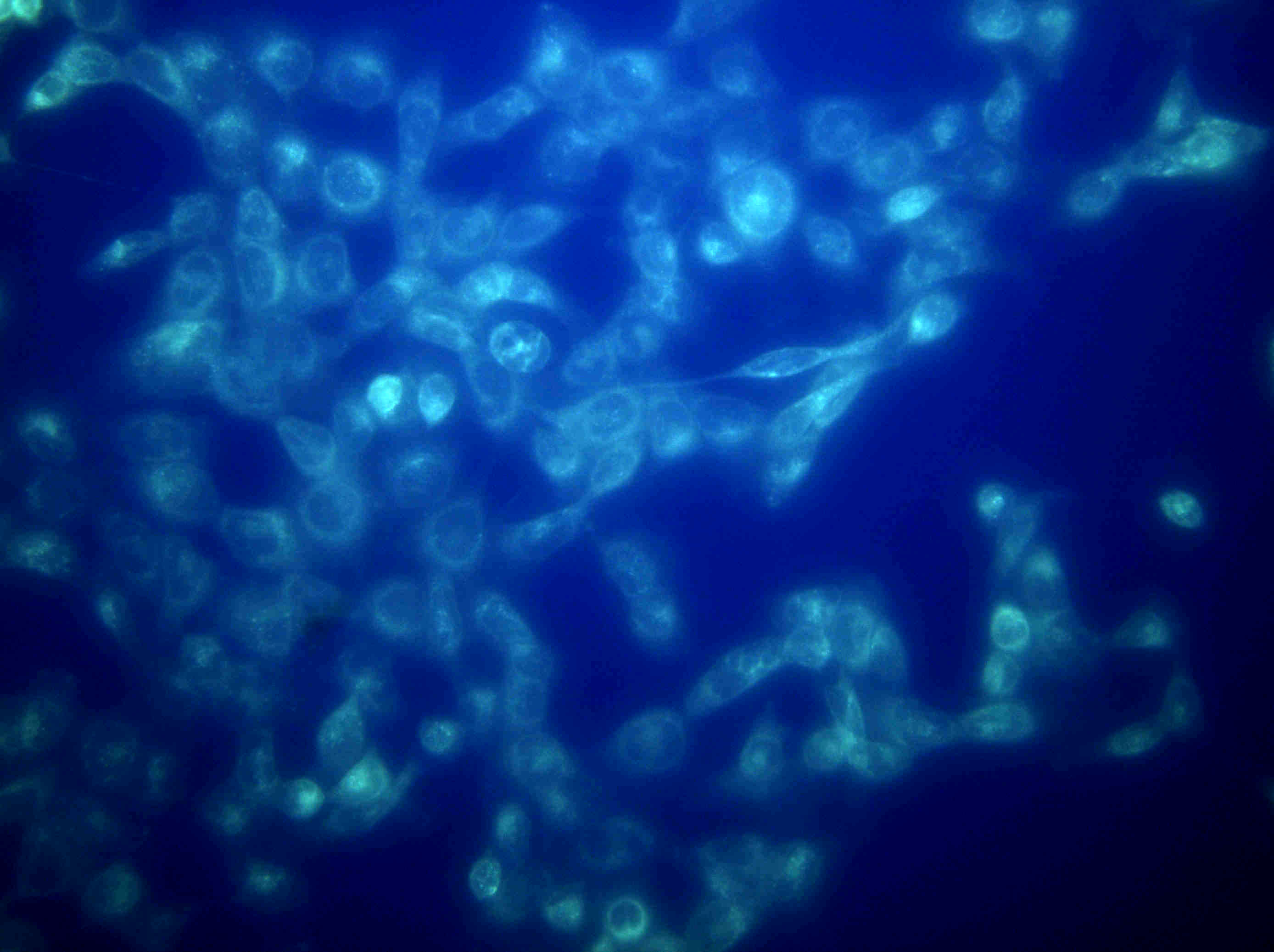

Supplement: Supplementary file 1 [file molecules-29-02919-s001.zip › Supplementary folder 1/0,2 treatment/Figure S2B.JPG]

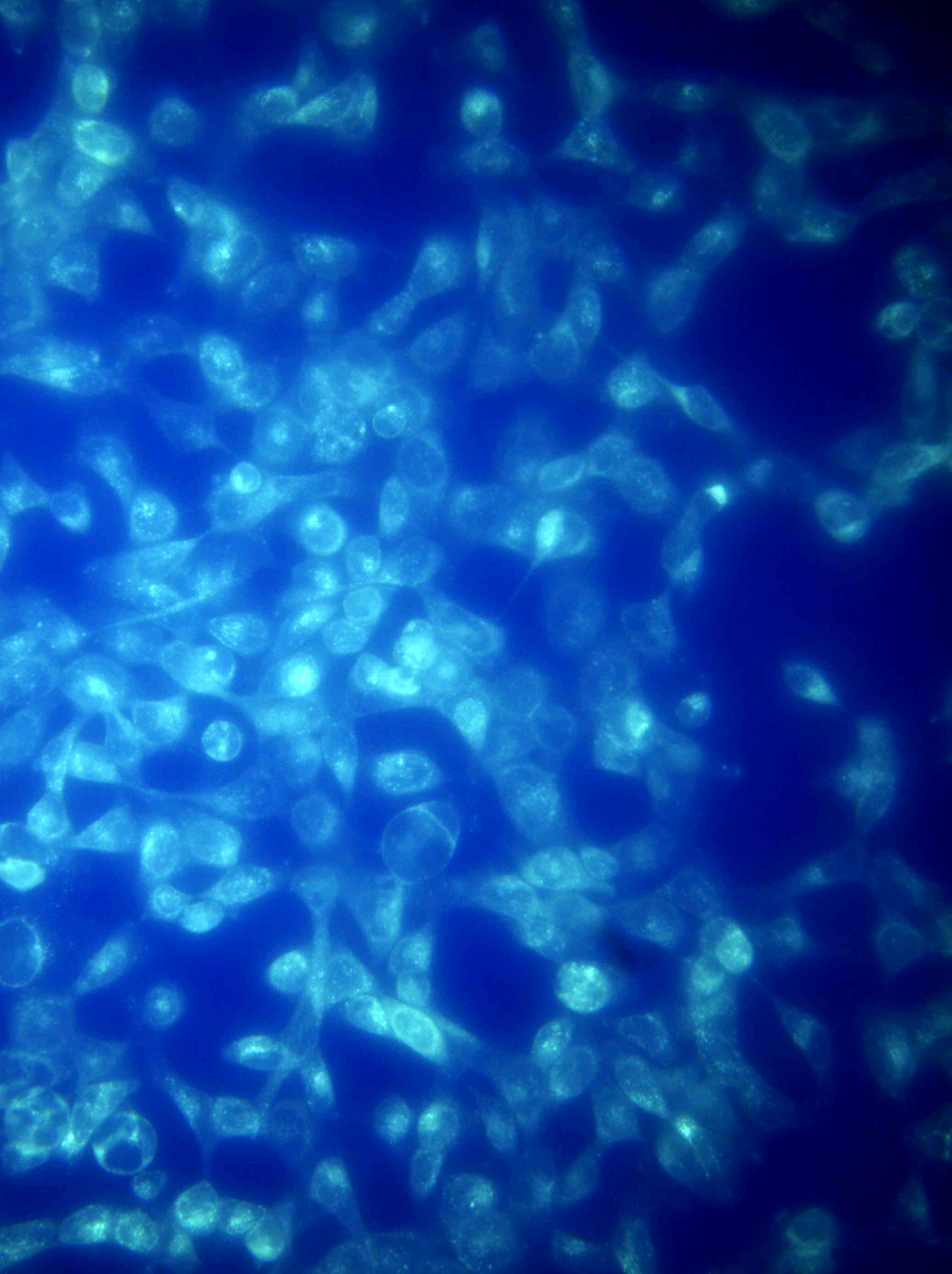

Supplement: Supplementary file 1 [file molecules-29-02919-s001.zip › Supplementary folder 1/0,2 treatment/Figure S3B.JPG]

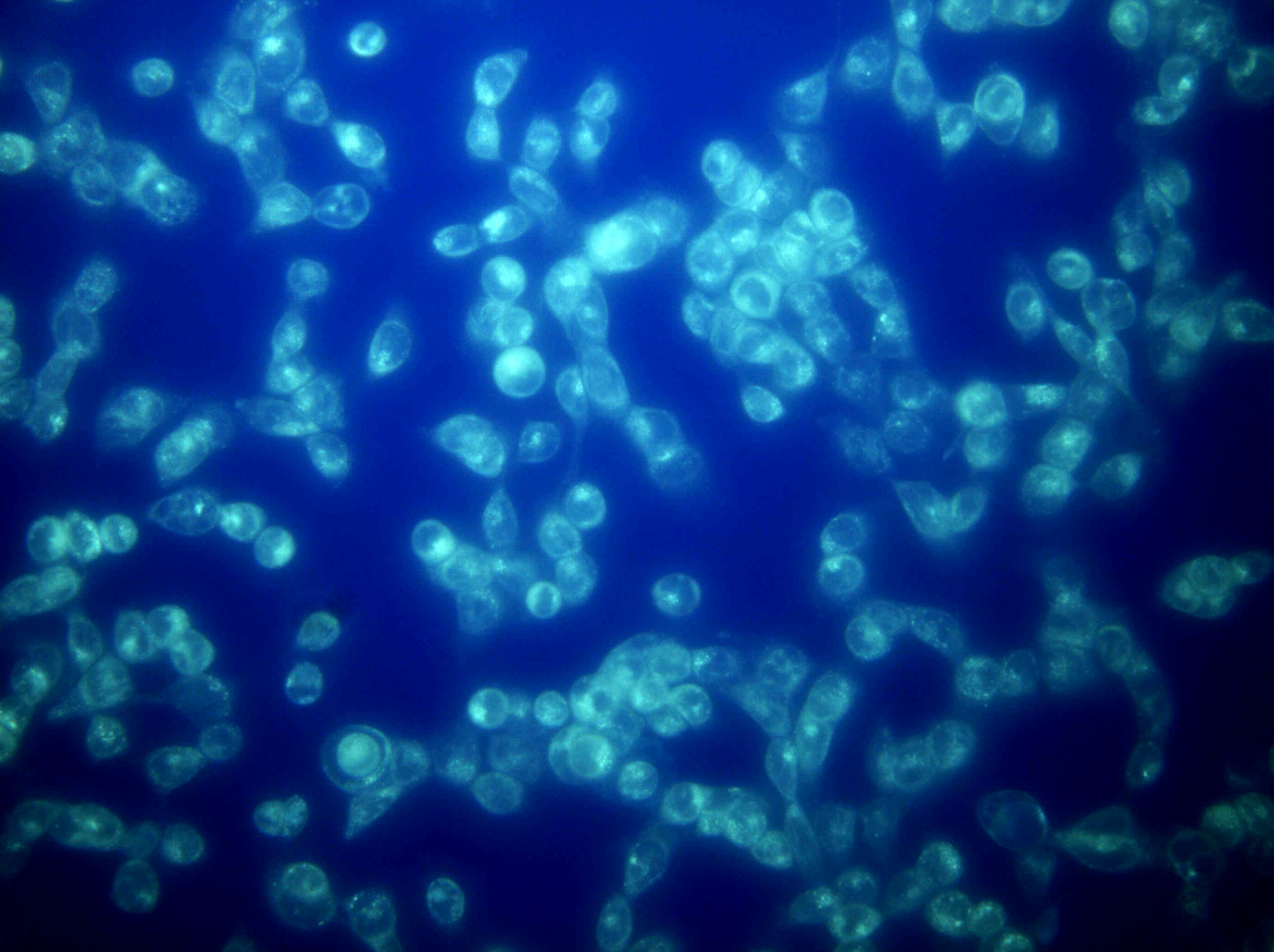

Supplement: Supplementary file 1 [file molecules-29-02919-s001.zip › Supplementary folder 1/0,4 treatment/Figure S1C.JPG]

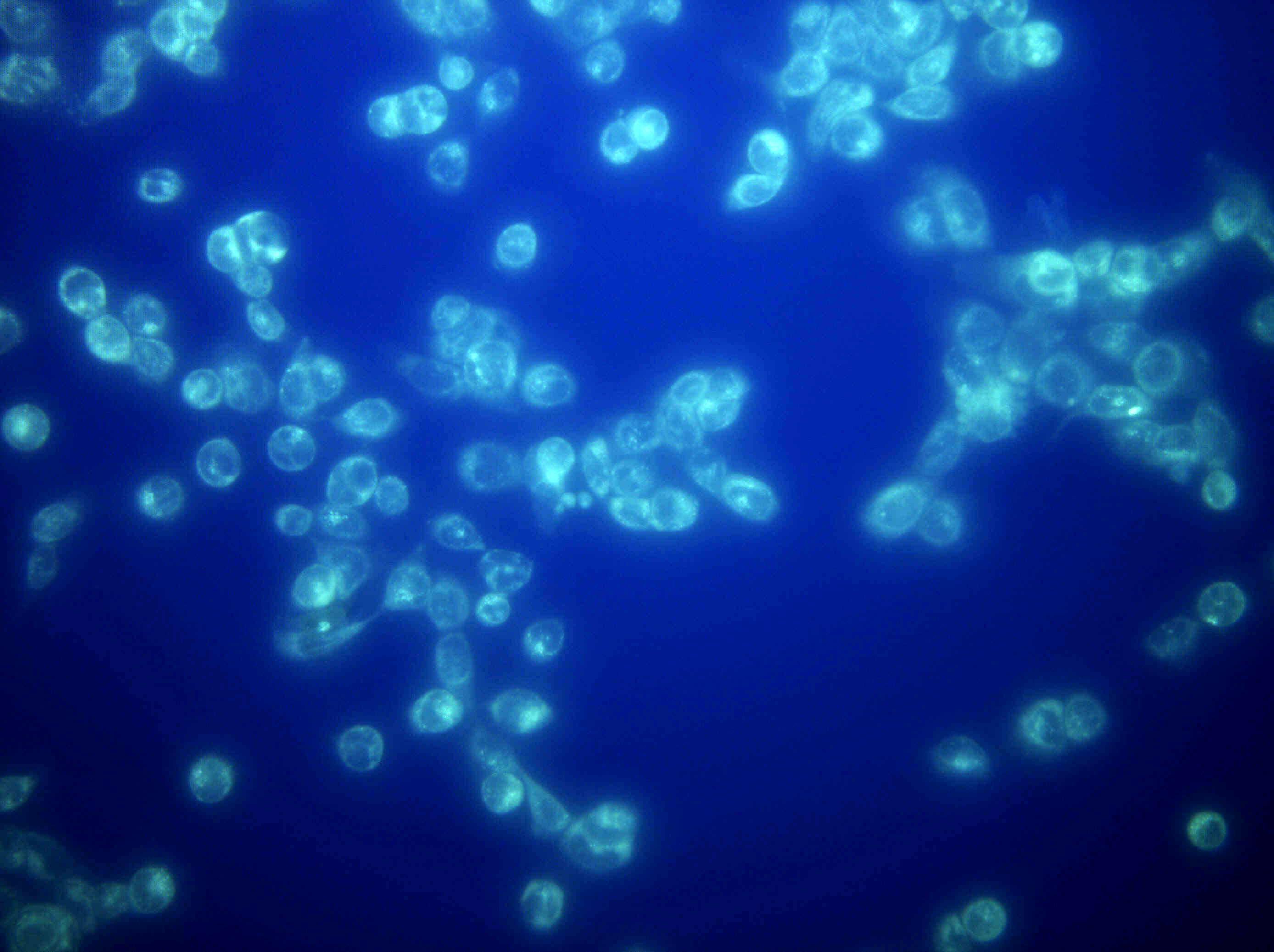

Supplement: Supplementary file 1 [file molecules-29-02919-s001.zip › Supplementary folder 1/0,4 treatment/Figure S2C.JPG]

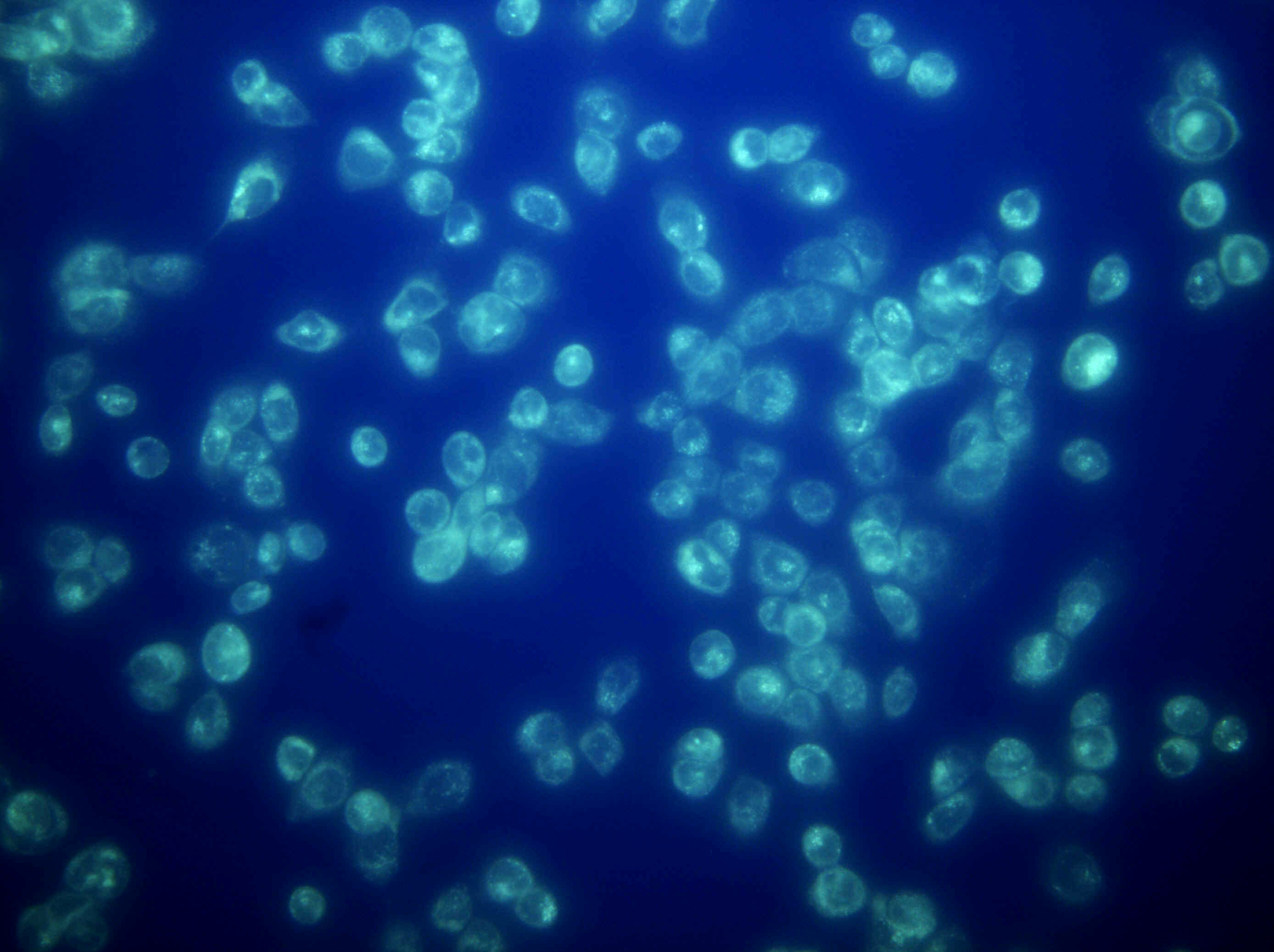

Supplement: Supplementary file 1 [file molecules-29-02919-s001.zip › Supplementary folder 1/0,4 treatment/Figure S3B.JPG]

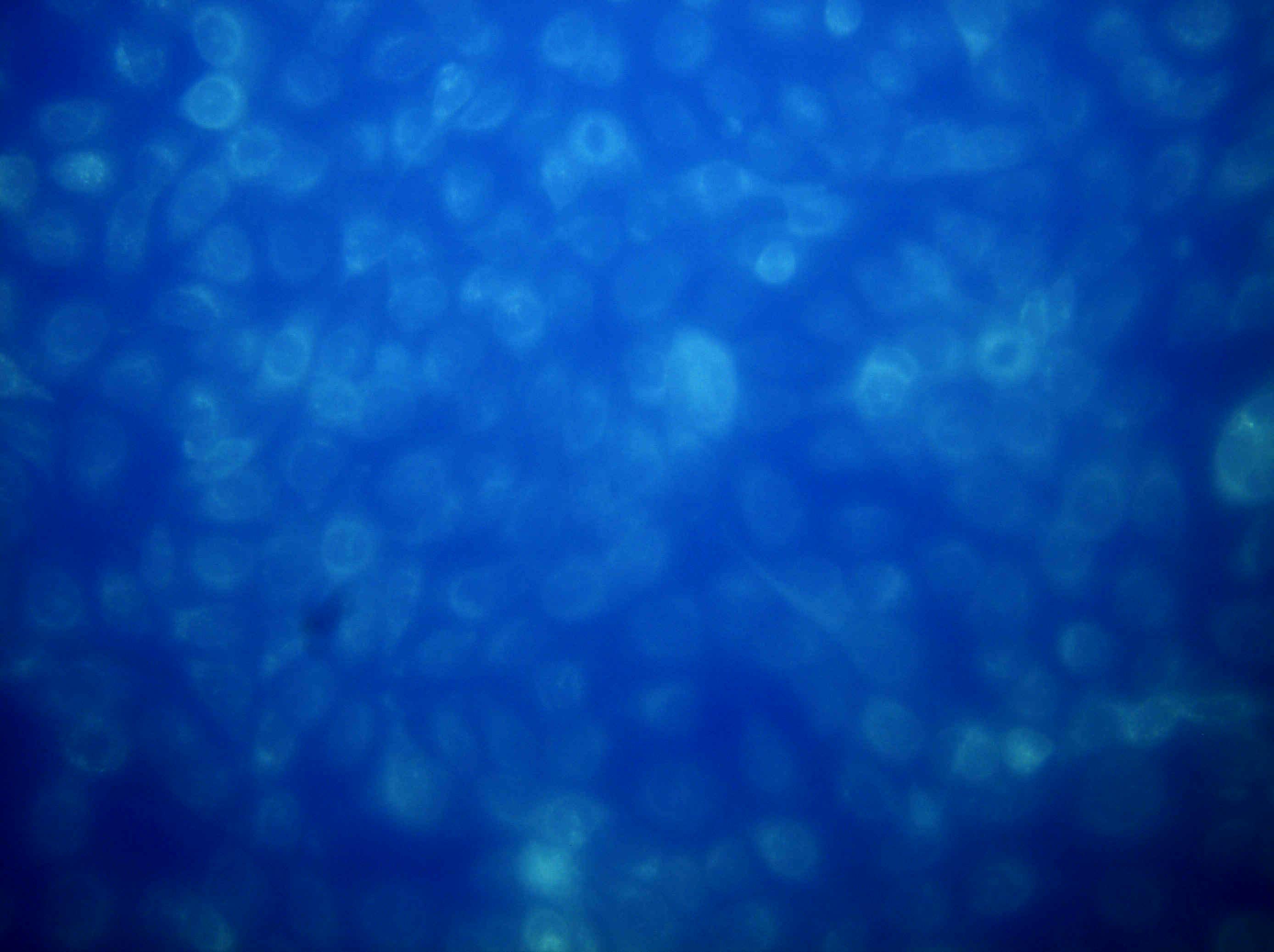

Supplement: Supplementary file 1 [file molecules-29-02919-s001.zip › Supplementary folder 1/Negative control/Figure S1E.jpg]

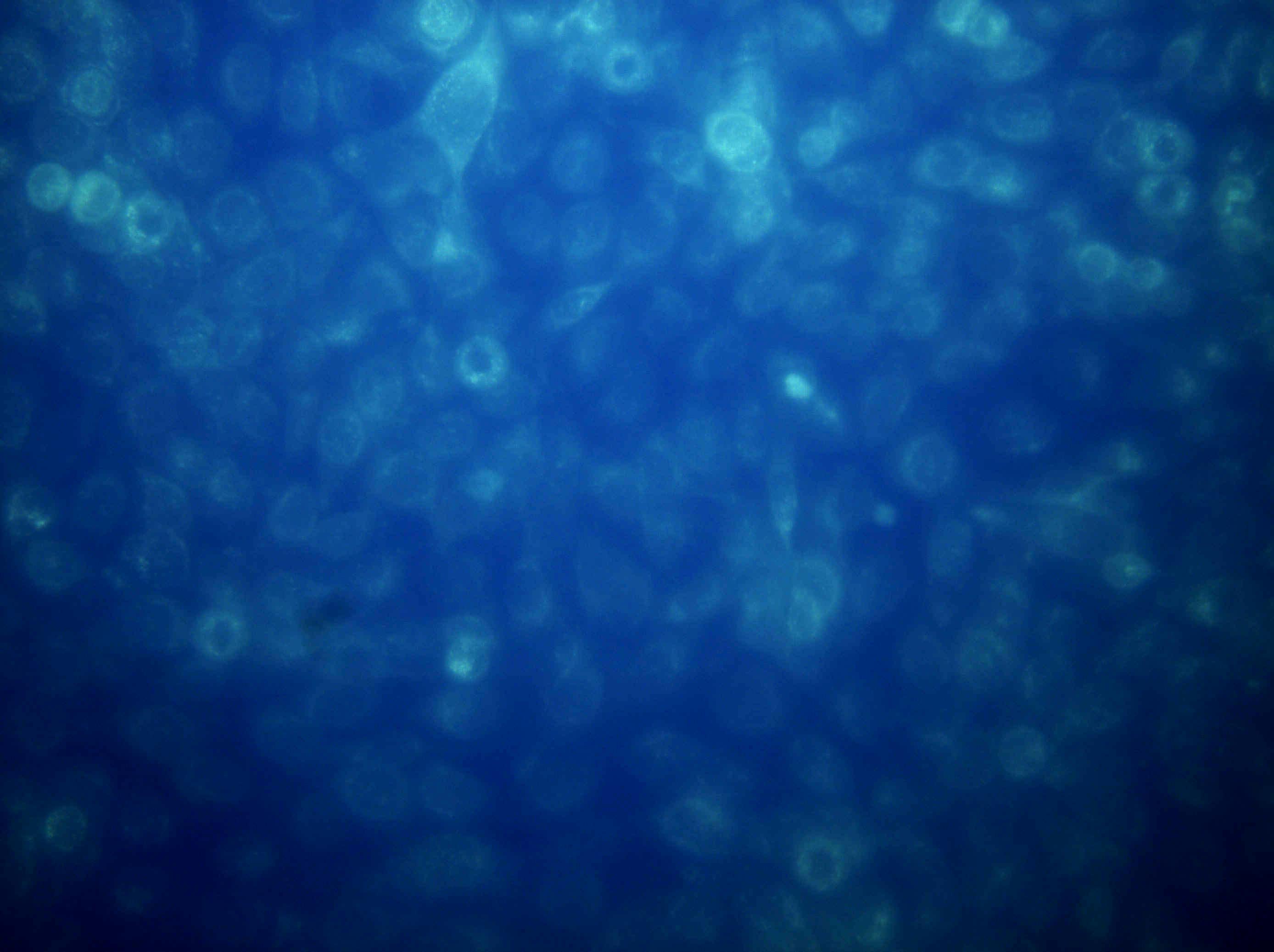

Supplement: Supplementary file 1 [file molecules-29-02919-s001.zip › Supplementary folder 1/Negative control/Figure S2E.jpg]

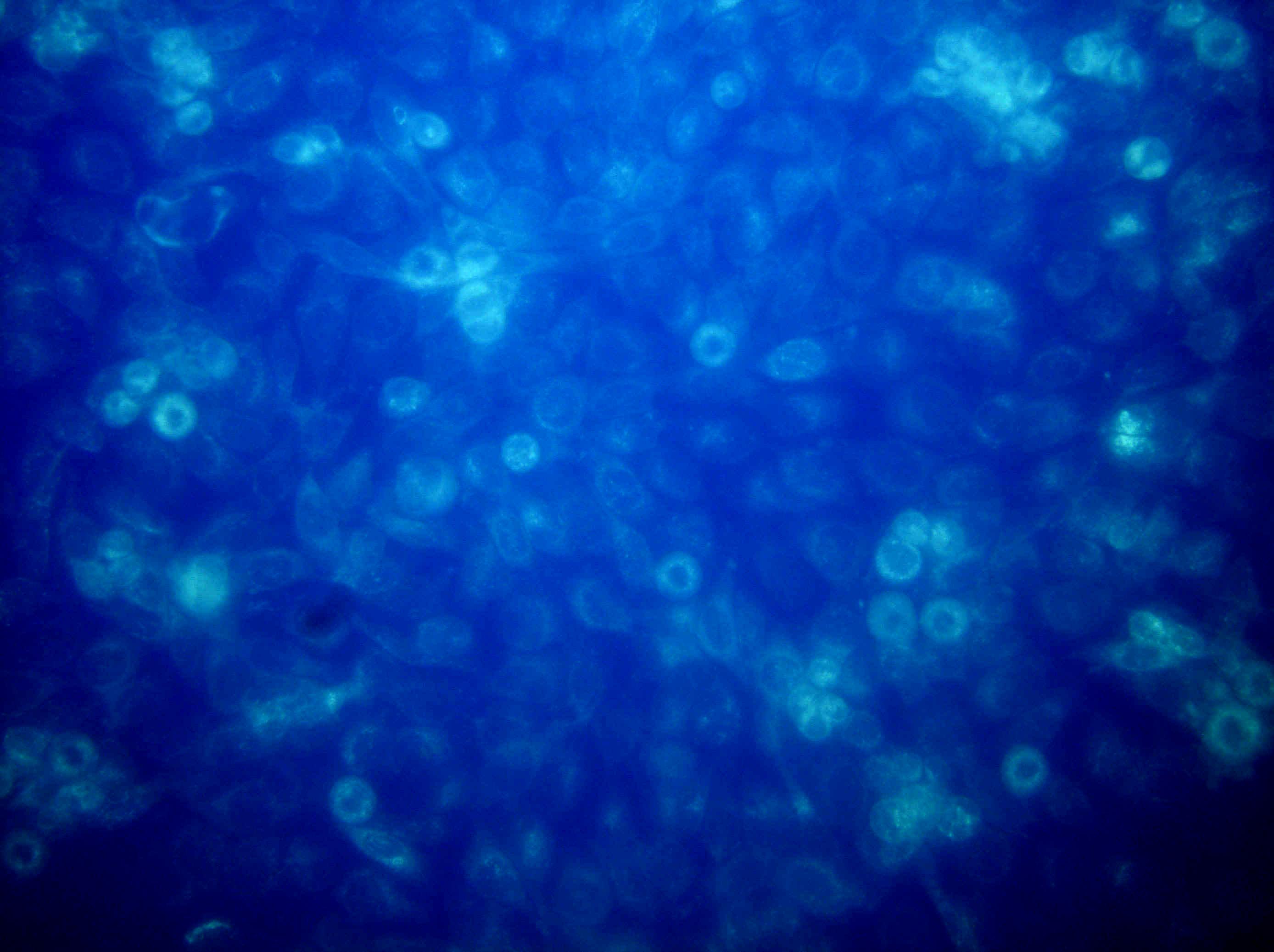

Supplement: Supplementary file 1 [file molecules-29-02919-s001.zip › Supplementary folder 1/Negative control/Figure S3E.jpg]

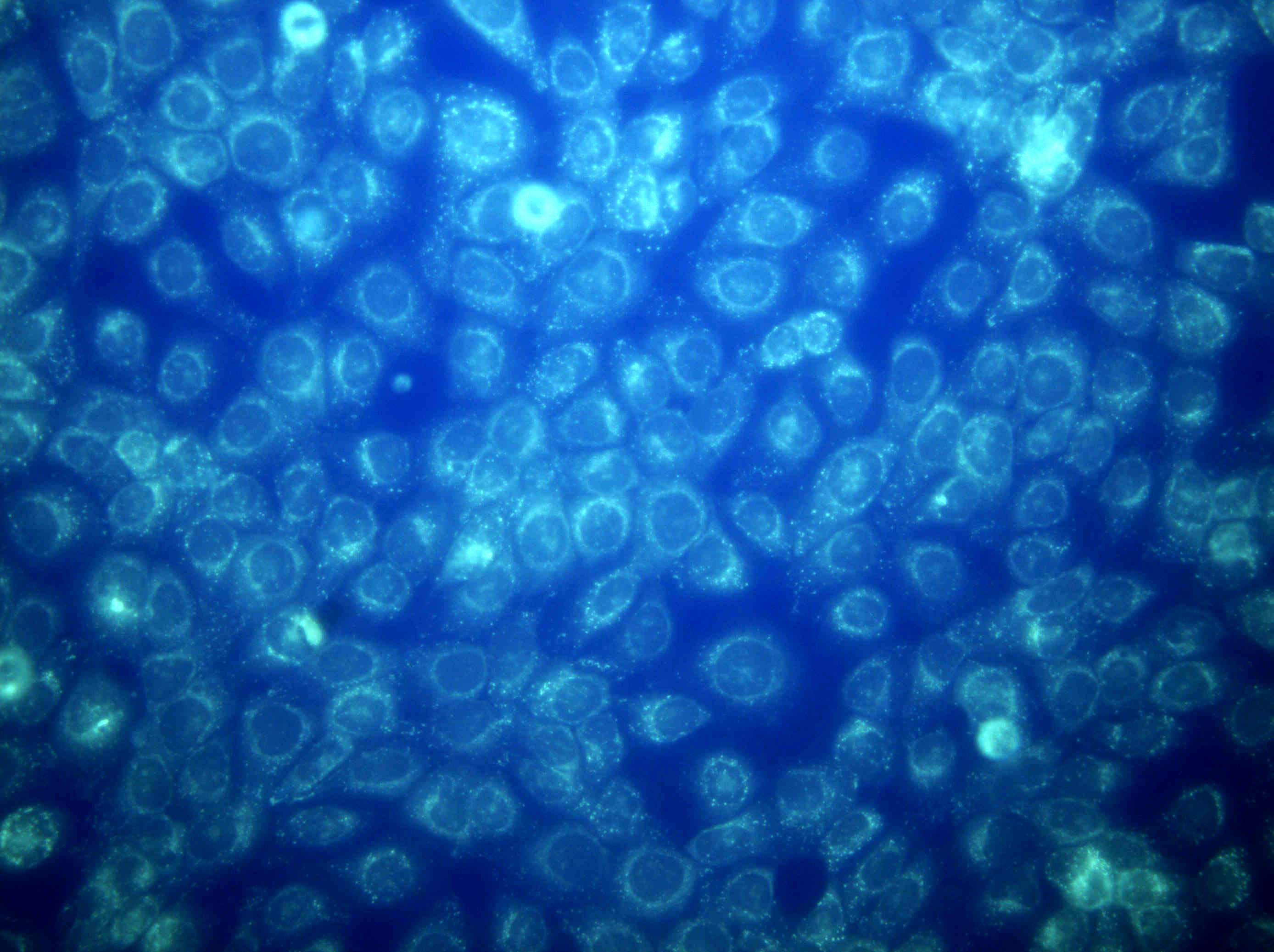

Supplement: Supplementary file 1 [file molecules-29-02919-s001.zip › Supplementary folder 1/Positive control/Figure S1F.JPG]

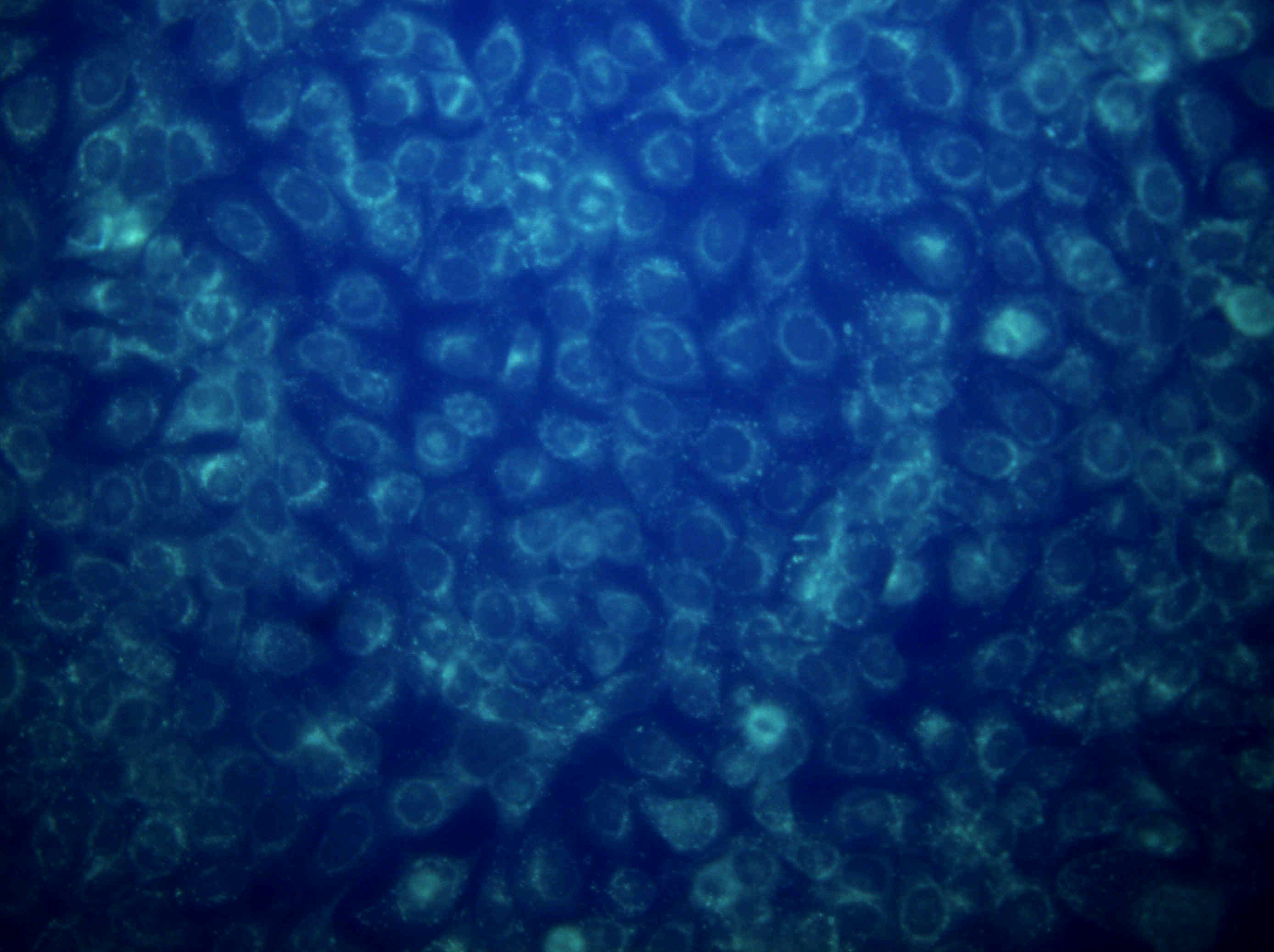

Supplement: Supplementary file 1 [file molecules-29-02919-s001.zip › Supplementary folder 1/Positive control/Figure S2F.JPG]

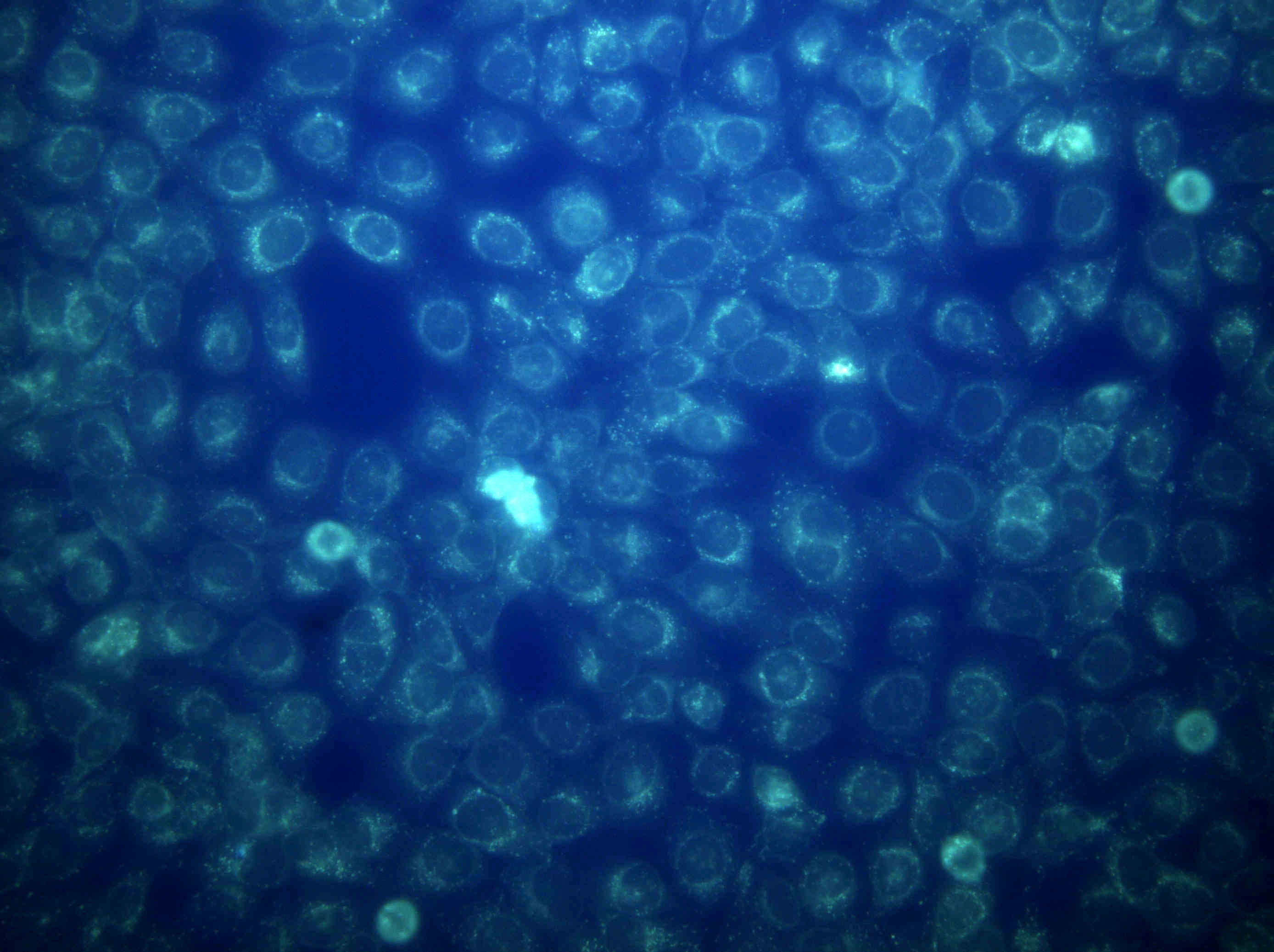

Supplement: Supplementary file 1 [file molecules-29-02919-s001.zip › Supplementary folder 1/Positive control/Figure S3F.JPG]
